# Bridging structure and function: A model of sequence learning and prediction in primary visual cortex

**DOI:** 10.1371/journal.pcbi.1006187

**Published:** 2018-06-05

**Authors:** Christian Klos, Daniel Miner, Jochen Triesch

**Affiliations:** 1 Frankfurt Institute for Advanced Studies, Frankfurt am Main, Germany; 2 Neural Network Dynamics and Computation, Institute of Genetics, University of Bonn, Bonn, Germany; Research Center Jülich, GERMANY

## Abstract

Recent experiments have demonstrated that visual cortex engages in spatio-temporal sequence learning and prediction. The cellular basis of this learning remains unclear, however. Here we present a spiking neural network model that explains a recent study on sequence learning in the primary visual cortex of rats. The model posits that the sequence learning and prediction abilities of cortical circuits result from the interaction of spike-timing dependent plasticity (STDP) and homeostatic plasticity mechanisms. It also reproduces changes in stimulus-evoked multi-unit activity during learning. Furthermore, it makes precise predictions regarding how training shapes network connectivity to establish its prediction ability. Finally, it predicts that the adapted connectivity gives rise to systematic changes in spontaneous network activity. Taken together, our model establishes a new conceptual bridge between the structure and function of cortical circuits in the context of sequence learning and prediction.

## Author summary

A central goal of Neuroscience is to understand the relationship between the structure and function of brain networks. Of particular interest are the circuits of the neocortex, the seat of our highest cognitive abilities. Here we provide a new link between the structure and function of neocortical circuits in the context of sequence learning. We study a spiking neural network model that self-organizes its connectivity and activity via a combination of different plasticity mechanisms known to operate in cortical circuits. We use this model to explain various findings from a recent experimental study on sequence learning and prediction in rat visual cortex. Our model reproduces the changes in activity patterns as the animal learns the sequential pattern of visual stimulation. In addition, the model predicts what stimulation-induced structural changes underlie this sequence learning ability. Finally, the model also predicts how the adapted network structure influences spontaneous network activity when there is no visual stimulation. Hence, our model provides new insights about the relationship between structure and function of cortical circuits.

## Introduction

The ability to predict the future is a fundamental challenge for cortical circuits. At the heart of prediction is the capacity to learn sequential patterns, i.e., the ability for sequence learning. Recent experiments have shown that even early sensory cortices such as rat primary visual cortex are capable of sequence learning [1]. Specifically, Xu et al. [1] have shown that the visual cortex can learn a repeated spatio-temporal stimulation pattern in the form of a light spot moving across a portion of the visual field. Intriguingly, when only the start location of the sequence is stimulated by the light spot after learning, the network will anticipate the continuation of the sequence as revealed by its spiking activity. While these results are remarkable, the cellular basis of this ability has remained elusive. A natural candidate for a cellular mechanism of sequence learning is spike-timing dependent plasticity (STDP) [2, 3]. Theoretical work has suggested that the inherent temporal asymmetry of STDP seems ideally suited for learning temporal sequences and storing them into the structure of cortical circuits [4–8]. At present, it is still unknown, however, exactly how such sequence memories are stored in real cortical circuits and how they become reflected in the structure of these circuits. Yet, a number of generic structural features of cortical circuits have been established in recent years. Among them is the lognormal-like distribution of synaptic efficacies between excitatory neurons [9–13], the distance-dependence of synaptic connection probabilities [12, 14], and an abundance of bidirectional connections between excitatory neurons [12, 15]. While recent theoretical studies have successfully modeled the origins of these generic structural features of cortical circuits, there is currently no unified model that explains both the emergence of structural features of cortical circuits and their sequence learning abilities. Here we present such a model and therefore establish a new conceptual bridge between the structure and function of cortical circuits.

Our model is a recurrently connected network of excitatory and inhibitory spiking neurons endowed with STDP, combined with a form of structural plasticity that creates new synapses at a low rate and destroys synapses whose efficacies have fallen below a threshold, as well as several homeostatic plasticity mechanisms. We used this network to model recent experiments on sequence learning in rat primary visual cortex [1]. The model successfully captured how multi-unit activity is changing during learning and explained these changes on the basis of STDP and the other plasticity mechanisms adapting the circuit during learning. It additionally demonstrated how homeostatic mechanisms prevent the runaway connection growth and unstable overlearning [16–19] that otherwise tends to occur from STDP alone. Furthermore, the model predicted that the changes to the network during learning also alter spontaneous activity patterns in systematic ways leading to an increased probability of spontaneous sequential activation. Finally, the model also captured the experimental finding that the training effect is only short-lasting. In sum, we present the first spiking neural network model that explains recent sequence learning data from rat primary visual cortex while also reproducing key structural features of cortical connectivity.

## Results

### Model summary

**Fig 1:**
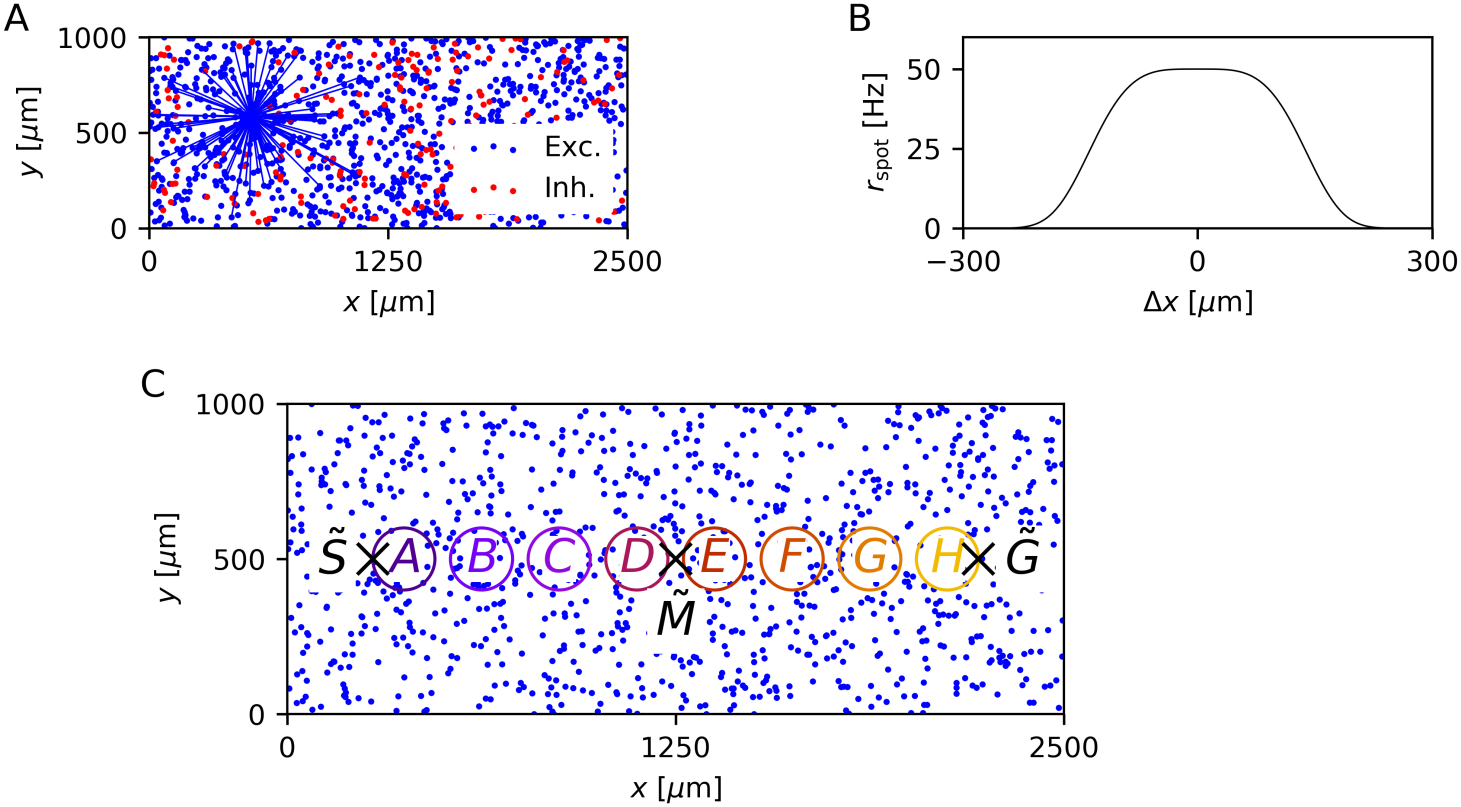
Network architecture and modeling of the study by Xu et al. [1] in the LIF-SORN. (A) Distribution of neurons on the 2D grid of one network instance. Blue lines show all connections projecting from the excitatory population to an example excitatory neuron after 500 s of simulation time. Connections spanning a larger distance are unlikely to exist, due to the distance dependent connection probability. (B) Cross-section of the rate *r*_spot_ of the Poissonian input spike trains with Δ*x* being the distance to the center of the spot. (C) Distribution of the excitatory neurons including the start point $\tilde{S}$, mid point $\tilde{M}$ and end point $\tilde{G}$ of the trajectory of the moving spot used in most experiments. The colored circles define the neurons that are pooled together for the analysis of the sequence learning ability.

The network model we used is a member of the class of self-organizing recurrent neural network (SORN) models (see e.g. [6, 20–23]). Specifically, we used the leaky integrate-and-fire SORN (LIF-SORN) introduced by Miner and Triesch [22]. The LIF-SORN is a model of a small section of L5 of the rodent cortex. Here, we used a version of it consisting of *N*^*E*^ = 1000 excitatory and *N*^I^ = 0.2 × *N*^E^ = 200 inhibitory leaky integrate-and-fire neurons with conductance based synapses and Gaussian membrane noise. The neurons are placed randomly on a 2500 μm × 1000 μm grid (Fig 1A) and their connectivity is distance-dependent, meaning a neuron is more likely to form a connection with a neuron nearby than with a remote neuron. While the weights of connections involving inhibitory neurons are fixed, recurrent excitatory connections are subject to a set of biologically motivated plasticity mechanisms. Exponential STDP with an asymmetric time window endows the network with the ability to learn correlations in external input. It is complemented by a form of structural plasticity (SP), which creates new and prunes weak connections. The network dynamics are stabilized by three additional plasticity mechanisms. First, synaptic normalization (SN) keeps the total incoming weight for each excitatory neuron constant. Second, intrinsic plasticity (IP) regulates the threshold potential of each excitatory neuron to counteract overly high or low firing rates. Third, short-term plasticity (STP) facilitates or impedes signal transmission along a specific connection based on the firing history of the presynaptic neuron.

Using this network model, we replicated the study by Xu et al. [1]. In this experiment, a multielectrode array was inserted in the primary visual cortex of rats and the receptive field of each channel was determined. The rats were then presented with a bright light spot, which was moved from a start point $\tilde{S}$ to an end, or goal, point $\tilde{G}$ along the distribution of receptive fields. The effect of this conditioning was assessed by measuring the responses to different kinds of cues of the full motion sequence. Xu et al. [1] investigated both awake and anesthetized rats, but unless noted otherwise, we compared our results to the results from awake animals. In the LIF-SORN, we modeled the movement of the light spot by sweeping a spot from $x_{\tilde{\text{S}}}={\left(375\;\mu \text{m},\;500\;\mu \text{m}\right)}^{\text{T}}$ to $x_{\tilde{\text{G}}}={\left(2125\;\mu \text{m},\;500\;\mu \text{m}\right)}^{\text{T}}$. The amplitude of this spot at the position $x_{n_{\text{e}}}$ of an excitatory neuron *n*_e_ represents the rate $r_{\text{spot}}\left(x_{n_{\text{e}}},t\right)$ of external Poissonian spike trains, which this neuron receives (Fig 1B). Furthermore, we approximated recording with a multielectrode array by introducing clusters of excitatory neurons. These clusters are located between $x_{\tilde{\text{S}}}$ and $x_{\tilde{\text{G}}}$ and are named *A* to *H* according to their distance to $x_{\tilde{\text{S}}}$ (Fig 1C). For the analysis of the sequence learning task, we only considered the activity of these clusters, which we defined to be the pooled spikes of the neurons part of a cluster. See the Materials and methods section for a more detailed description of our model.

### Basic network properties

**Fig 2:**
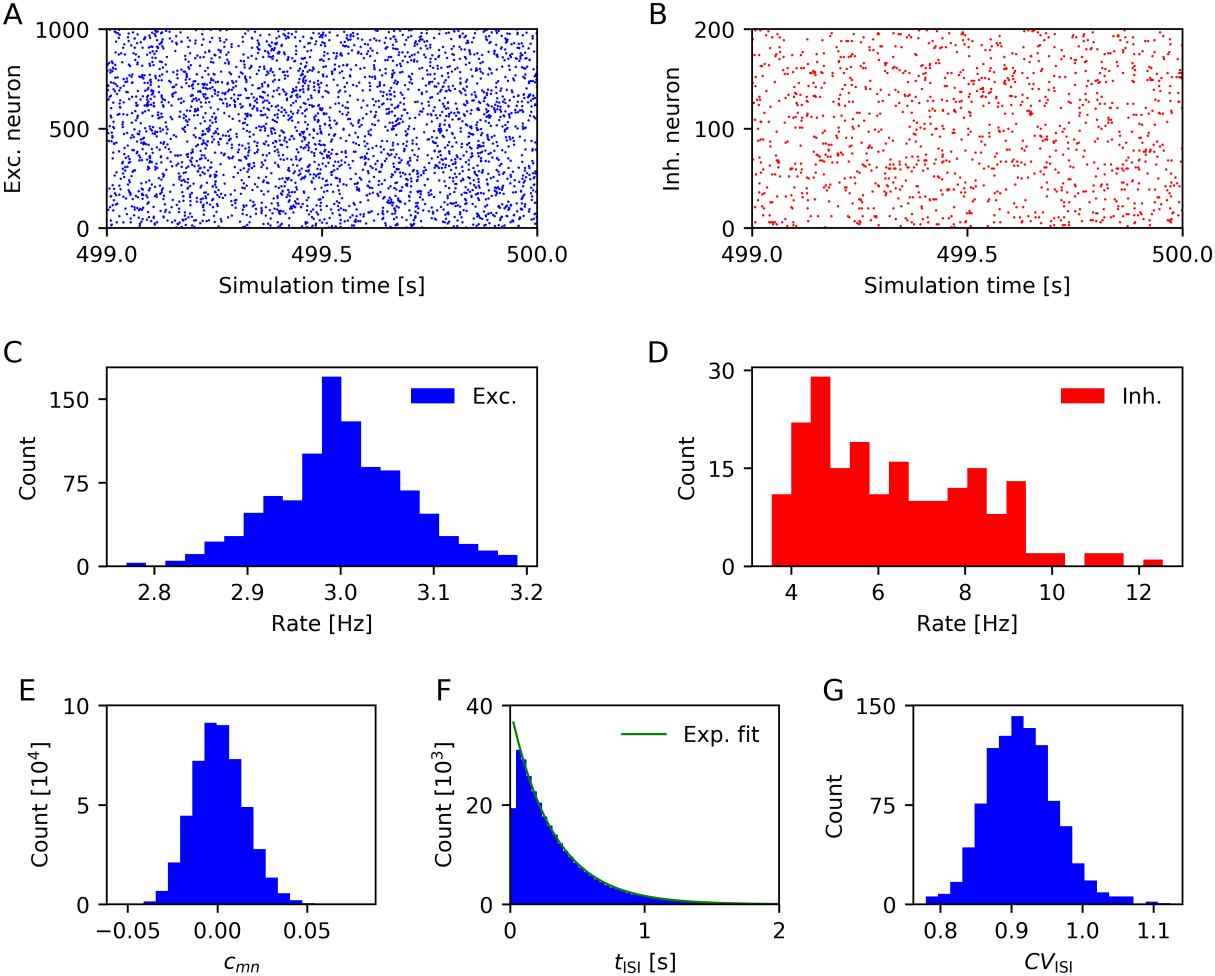
Characterization of spiking activity (based on activity in the time window 400s–500s). (A) Spike trains of excitatory neurons. (C) Firing rate distribution of excitatory neurons. (B,D) Same as (A,C) for inhibitory neurons. (E) Distribution of pairwise correlation coefficients of the excitatory population, computed for time bins of 20 ms duration. (F) Interspike interval distribution of all excitatory neurons (Exponential function is fitted to the data points with *t*_ISI_ > 50 ms). (G) Distribution of coefficients of variation of the interspike interval distribution of each excitatory neuron. Data from a single network instance.

To get an impression of the behavior of the LIF-SORN, we simulated the network for 500 s solely under the influence of the background noise, i.e., without external input. Thereby we also showed that it exhibits some basic properties of both the activity and connectivity in biological neural networks. We began by analyzing the spiking activity after an initial equilibration phase (Fig 2).

Both excitatory and inhibitory neurons seemed to exhibit unstructured firing (Fig 2A and 2B) with the excitatory neurons firing with frequencies distributed closely around 3 Hz due to the IP (Fig 2C) and inhibitory neurons firing with roughly twice this frequency (Fig 2D). The activity of the cortex in the absence of external stimuli is often assumed to lie in a regime of asynchronous irregular spiking. Synchrony refers, in this context, to the joint spiking of a set of neurons. Biological data shows that population level activity in the cortex is highly asynchronous [24, 25]. Mostly, the pairwise correlation coefficient is used to quantify synchrony (see e.g. [26]). The pairwise correlation coefficient between a neuron *m* and *n* is defined as

$$\begin{array}{c} c_{mn}=\frac{\text{cov}(C_{m},C_{n})}{\sqrt{\text{Var}\left(C_{m}\right)\text{Var}\left(C_{n}\right)}}\;, \end{array}$$ (1)

where *C*_*m*_ is the time series of spike counts of neuron *m* within successive time bins. Fig 2E shows the distribution of pairwise correlation coefficients of all disjoint pairs of excitatory neurons in the LIF-SORN for time bins of 20 ms duration. The pairwise correlation coefficients were closely distributed around zero, implying a low level of synchrony in the LIF-SORN. When varying the duration of the time bins, the mean of the distribution of pairwise correlation coefficients stayed close to zero while its width increased (decreased) with increasing (decreasing) duration of the time bins.

Regularity refers to the variability of the spiking of individual neurons. In the cortex, this spiking is highly irregular and can, apart from the refractory period, often be quite accurately described by a Poisson process [27], in which the interspike intervals follow an exponential distribution with a coefficient of variation equal to unity. We found that interspike intervals of excitatory neurons in the LIF-SORN were approximately exponentially distributed with a distortion caused by the refractory period (Fig 2F) and that the coefficients of variation were generally close to one (Fig 2G), indicating irregular spiking.

**Fig 3:**
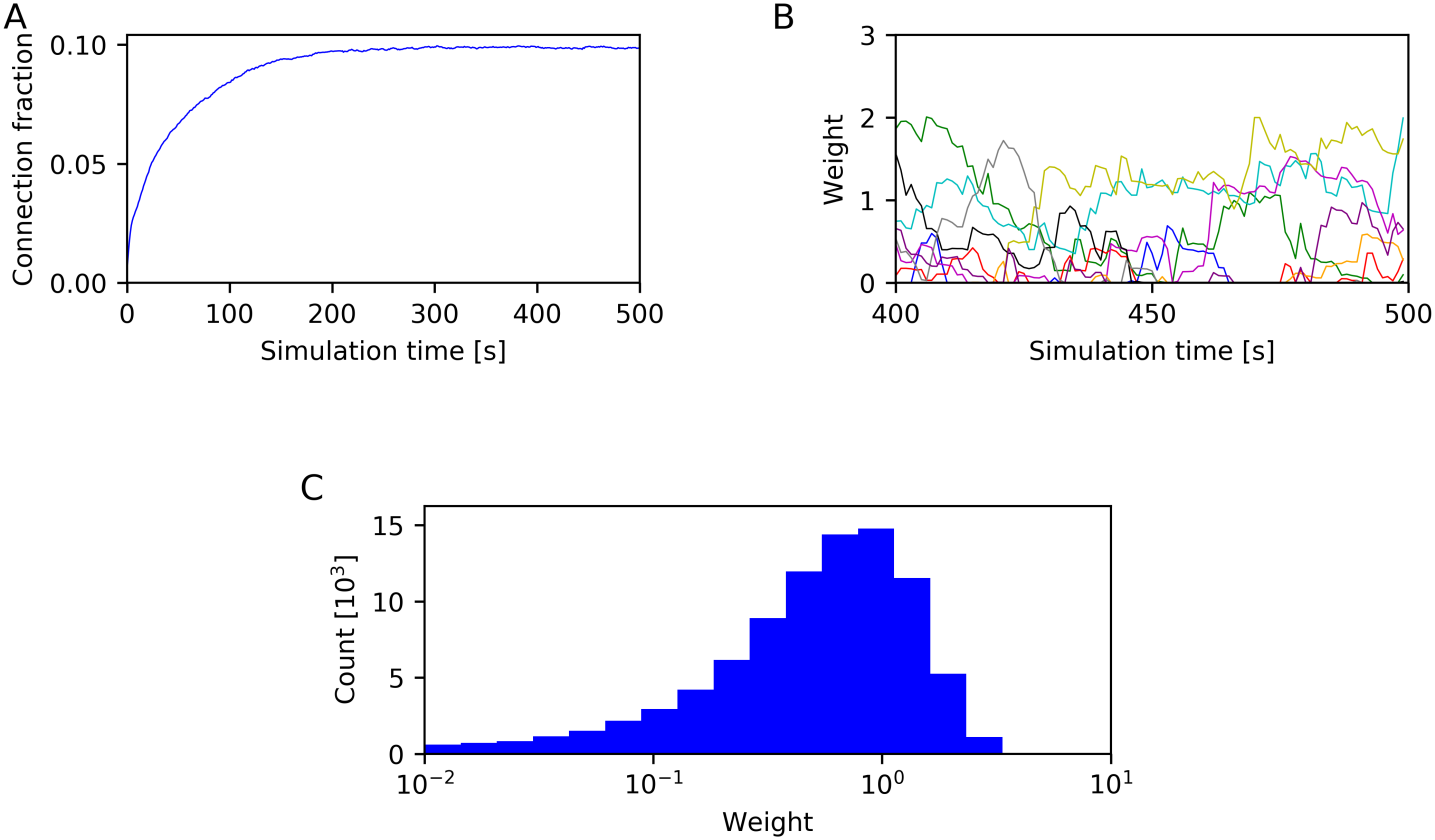
Characterization of network connectivity. (A) Connection fraction of recurrent excitatory connections. (B) Time course of the weights of 10 randomly selected recurrent excitatory connections after the connection fraction has stabilized. (C) Distribution of the weights of existing recurrent excitatory connections, determined after 500 s of simulation time. Data from a single network instance.

Next, we considered the structural properties of the LIF-SORN. The LIF-SORN was initialized without recurrent excitatory connections, but due to SP, these connections grew for about 200 s, as can be seen in Fig 3A. Afterwards the pruning rate of existing synapses approached the growth rate of new synapses and the network entered a stable phase in which the connection fraction of excitatory connections did not change anymore. The values of individual weights were, on the other hand, still fluctuating (Fig 3B). This constant change was also found in biological networks [13]. Additionally, the excitatory weights assumed an approximately lognormal-like distribution (Fig 3C) as observed in cortical circuits [9–13]. We also converted the connection weights in approximate amplitudes of the corresponding postsynaptic potentials (PSP). In excitatory neurons, the mean excitatory PSP (EPSP) amplitude was 0.72 mV and the mean inhibitory PSP (IPSP) amplitude was 0.96 mV and in inhibitory neurons, the mean EPSP amplitude was 0.74 mV and the mean IPSP amplitude was 0.94 mV. These values lie within the experimentally observed range [11, 12, 28]. See S1 Appendix for a description of how this conversion was done and figures of the distribution of PSP amplitudes and their ratios.

Taken together, the LIF-SORN displayed key features of both the activity and connectivity in cortical circuits. Besides the here mentioned structural properties, the LIF-SORN has also already been shown to reproduce more complex properties of cortical wiring, namely the overrepresentation of bidirectional connections and certain triangular graph motifs compared to a random network and various aspects of synaptic dynamics [22].

### Cue-triggered sequence recall

To investigate the sequence learning ability of the LIF-SORN, we employed a similar test paradigm as Xu et al. [1]. That means we trained the network with the moving spot as described above and tested it by stimulating the network with brief flashes of the spot at the start point $x_{\tilde{\text{S}}}={\left(375\;\mu \text{m},\;500\;\mu \text{m}\right)}^{\text{T}}$, the mid point $x_{\tilde{\text{M}}}={\left(1250\;\mu \text{m},\;500\;\mu \text{m}\right)}^{\text{T}}$ and the end point $x_{\tilde{\text{G}}}={\left(2125\;\mu \text{m},\;500\;\mu \text{m}\right)}^{\text{T}}$. This testing was performed before and after training and the responses to each of the stimuli after training were compared to their counterpart before training.

Specifically, our simulation protocol started with a growth phase of 400 s duration, to initialize a network that exhibits key features of cortical circuits. It followed a test phase, during which one of the cues, i.e. a brief flash of the spot at the start point, mid point or end point, was presented once every two seconds. This first test phase lasted 100 s, leading to a total of 50 repetitions. After this test phase, the network was given a short relaxation phase of 10 s such that its activity got back to base level. Afterwards, the full motion sequence was shown to the network in the training phase, which lasted 200 s. The sequence was also presented once every two seconds leading to a total of 100 repetitions. After another relaxation phase of 10 s, the simulation ended with another test phase, during which the same cue as in the first test phase was shown to the network. This second test phase lasted 100 s leading to a total of 50 repetitions.

**Fig 4:**
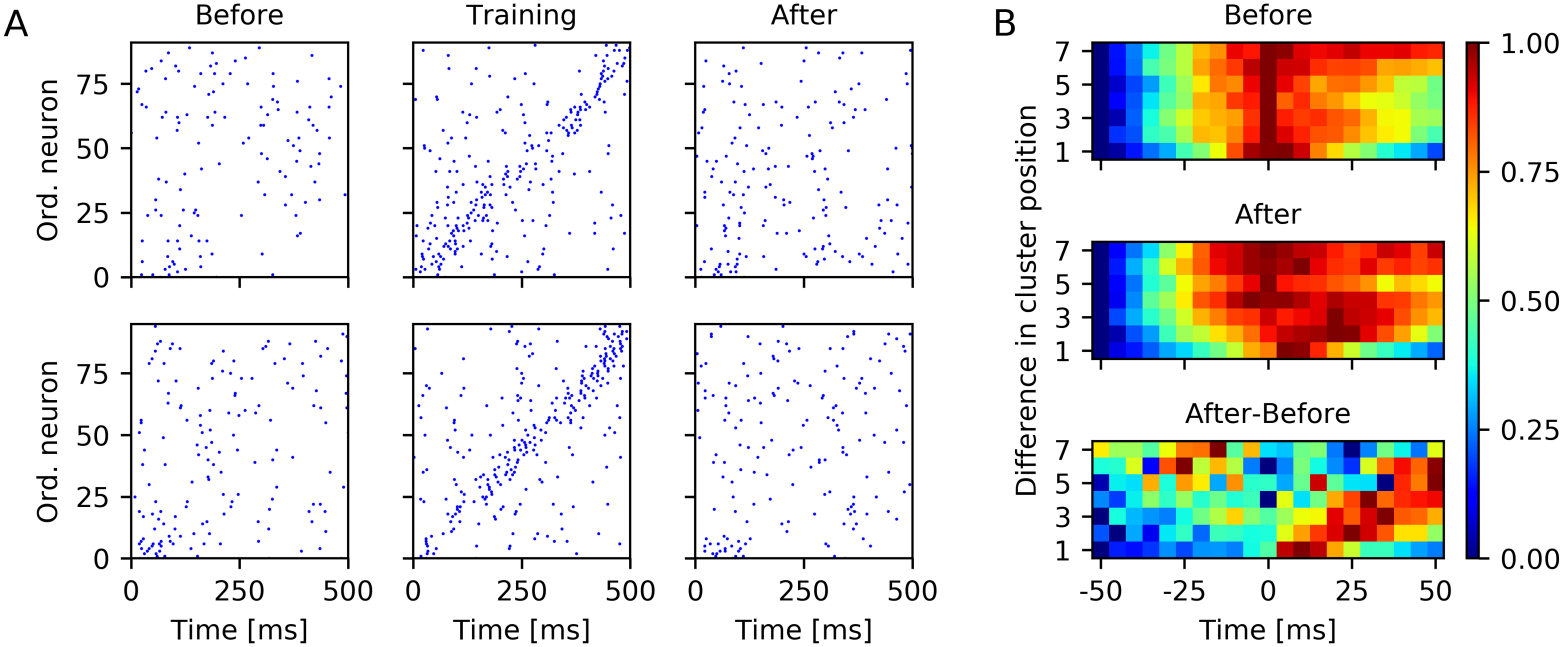
Cue-triggered sequence replay. (A) Example spike trains of neurons part of clusters in response to a brief flash of the light spot at $\tilde{S}$ before and after training and in response to the full motion sequence during training. Top and bottom row show spike trains for two different network instances. Neurons are ordered according to the projection of their location on the $\tilde{S}\to \tilde{G}$ axis. (B) Normalized pairwise cross-correlation between spikes of neurons part of clusters in response to a brief flash of the light spot at $\tilde{S}$ before (top) and after (middle) training and normalized difference between the cross-correlograms after and before training (bottom). Panel B shows results from 20 instances of the LIF-SORN.

The purpose of the presentation of the test cue at the start point was to examine if the network learned the sequential structure of the motion sequence. Fig 4A shows the spike trains of the neurons that are part of one of the clusters *A* to *H* during training in response to one presentation of the full motion sequence as well as before and after training in response to one trial of cue presentation at the start point. While the spiking was clearly sequential during training, such sequential spiking was much less pronounced in response to the test cue before and after learning. Additionally, the spiking was quite variable over trials and different networks. Similar results were found by Xu et al. [1].

A common method to assess the sequential spiking in animal studies of sequence replay is to compute the cross-correlation between pairs of spike trains [1, 29]. We performed such an analysis in a similar way as Xu et al. [1] by pooling the spikes for each cluster and than computing the correlation between these spike trains for each trial and for all cluster combinations. Therefor, we only considered spikes within the window 0 ms–500 ms relative to stimulus onset to minimize the impact of spontaneous activity. Next, we pooled the cross-correlations according to the corresponding difference in cluster position. This was done for the test phases before and after training. We then also took the difference between the resulting cross-correlograms and finally normalized each of the three cross-correlograms to the range between 0 and 1. This was done independently for each of the sets of cross-correlations corresponding to a specific difference in cluster position. Fig 4B shows the thereby obtained cross-correlograms, which qualitatively resembled the cross-correlograms obtained from rats [1] in that the correlation function took on higher values for positive time delays compared to negative time delays even before learning—an observation that can be linked to the spread of activity from $\tilde{S}$ towards $\tilde{G}$—and in that this rightward slant enhanced due to training. This increase of the correlation function for positive time delays indicated that the network indeed learned about the sequential structure of the motion sequence.

To quantify the cue-triggered sequence recall, we again adapted the analysis used by Xu et al. [1]. That is to say we pooled the spikes of all neurons for each cluster and calculated their rate by convolving them with a Gaussian filter with width *τ*_rate_ = 50 ms. We only considered spikes within the window 0 ms–500 ms relative to stimulus onset to minimize the impact of spontaneous activity and defined the firing time of a cluster as the first peak of its rate curve. Then we computed for each test trial the Spearman correlation coefficient between the firing times of the clusters and their location on the $\tilde{S}\to \tilde{G}$ axis. The Spearman correlation coefficient between two variables is defined as the Pearson correlation coefficient between the rank values of those variables. Hence, it measured how much the replay order resembled the training order of clusters.

**Fig 5:**
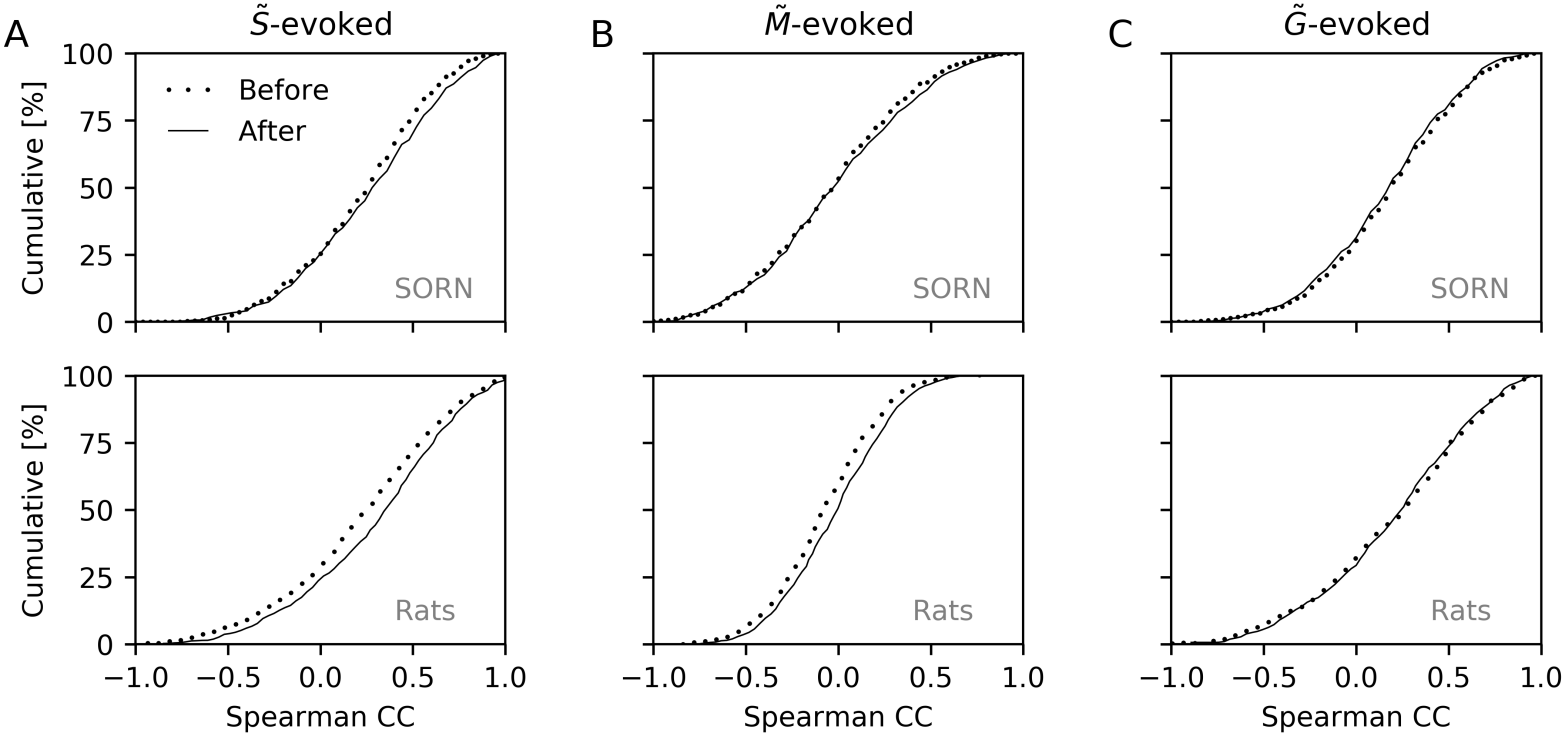
Analysis of cue-triggered sequence replay. (A) Top: Cumulative distribution of Spearman correlation coefficients when testing with a cue presented at $\tilde{S}$. The distribution is shifted to the right after (solid) compared to before (dotted) training. Bottom: Same as top but for results in rats [1]. (B,C) Same as A but for $\tilde{M}$-evoked (B) and $\tilde{G}$-evoked (C) responses. Top plots of all panels show results from 20 instances of the LIF-SORN. Bottom plots show approximate experimental data that were obtained from [1] using WebPlotDigitizer [30].

For the test phases with a cue at $\tilde{S}$, we found a significant rightward shift of the correlation coefficient distribution after learning with a change in mean from 0.26 to 0.30 (*P* = 7.9 × 10^−3^; Kolmogorov-Smirnov test) as shown in Fig 5A. Thus, there was enhanced sequential spiking after training compared to before training as found by Xu et al. [1], who observed a change in mean from 0.21 to 0.29 (*P* = 1.5 × 10^−3^; Kolmogorov-Smirnov test; Fig 5A).

The purpose of the presentation of the test cue at the mid point was to avoid having a rightward bias even before training. For that case, Xu et al. [1] also found a significant rightward shift of the correlation coefficient distribution with a change in mean from −0.08 to −0.02 (*P* = 1.3 × 10^−4^; Kolmogorov-Smirnov test; Fig 5B). In the LIF-SORN, we observed only a very small rightward shift, which wasn’t significant, however (change in mean from 0.0 to 0.02; *P* = 0.31; Kolmogorov-Smirnov test; Fig 5B).

The purpose of the presentation of the test cue at the end point was to examine if the cue-triggered replay was specific to the direction of the motion sequence. Thereby, Xu et al. [1] computed the Spearman correlation coefficients between the firing times of the clusters and their location on the $\tilde{G}\to \tilde{S}$ axis and found no significant shift in the correlation coefficient distribution (change in mean from 0.20 to 0.20, *P* = 0.59; Kolmogorov-Smirnov test; Fig 5C), indicating that the direction of replay was indeed specific to the direction of the motion sequence used during training. In the LIF-SORN, we found a small but not significant leftward shift (change in mean from 0.22 to 0.20, *P* = 0.53; Kolmogorov-Smirnov test; Fig 5C). The small leftward shift can be explained by the weakening of connections between the clusters pointing in the opposite direction of the motion sequence due to STDP, since this decreased the correlation between the activity of one cluster and a cluster located further along the $\tilde{G}\to \tilde{S}$ axis. Although Xu et al. [1] did not observe even a small leftward shift, it may still be that STDP was also responsible for the training effect in rats as the weakening of connections pointing in the opposite direction of the motion sequence could have been too small to have an observable effect. Furthermore, Xu et al. [1] showed that blocking NMDA receptors lead to the disappearance of the training effect indicating that some form of NMDA-dependent plasticity was indeed responsible for the training effect.

To test whether the training effect was restricted to a small region of V1 or if it was also apparent elsewhere in V1, Xu et al. [1] performed an experiment where, during training, the motion sequence was shifted orthogonal to the long axis of the recorded distribution of the receptive fields, i.e. orthogonal to the $\tilde{S}\to \tilde{G}$ axis. They neither found a significant shift in the correlation coefficient distribution for $\tilde{S}$-evoked nor for $\tilde{G}$-evoked responses and concluded that the effect of learning was indeed specific to the location of the motion sequence. As in the animal study, the LIF-SORN exhibited for this scenario no significant shift in the correlation coefficient distribution for both $\tilde{S}$-evoked and $\tilde{G}$-evoked responses (S1 Fig).

To examine if training with a dynamic stimulus is actually necessary to achieve a more distinctive sequence replay, two different experiments using a static stimulus during training were performed by Xu et al. [1]. In the first one, this stimulus was a flashed bar spanning the region between $\tilde{S}$ and $\tilde{G}$. In the second one, the stimulus used during training was just a briefly flashed spot at $\tilde{S}$. A significant shift in the correlation coefficient distribution was neither found for $\tilde{S}$-evoked nor for $\tilde{G}$-evoked responses. Again, the LIF-SORN also didn’t show significant training effects for these scenarios (S1 Fig).

### Training induces stripe-like connectivity

The activity of the LIF-SORN is determined by the input and its connectivity. In this section, we show how the training with a moving spot modulated the connectivity through the plasticity mechanisms. Therefore, we analyzed the weight matrix of the recurrent excitatory connections before and after training with the full motion sequence from $\tilde{S}$ to $\tilde{G}$. This allowed us to connect a large part of the results of the previous sections with the change in connectivity.

**Fig 6:**
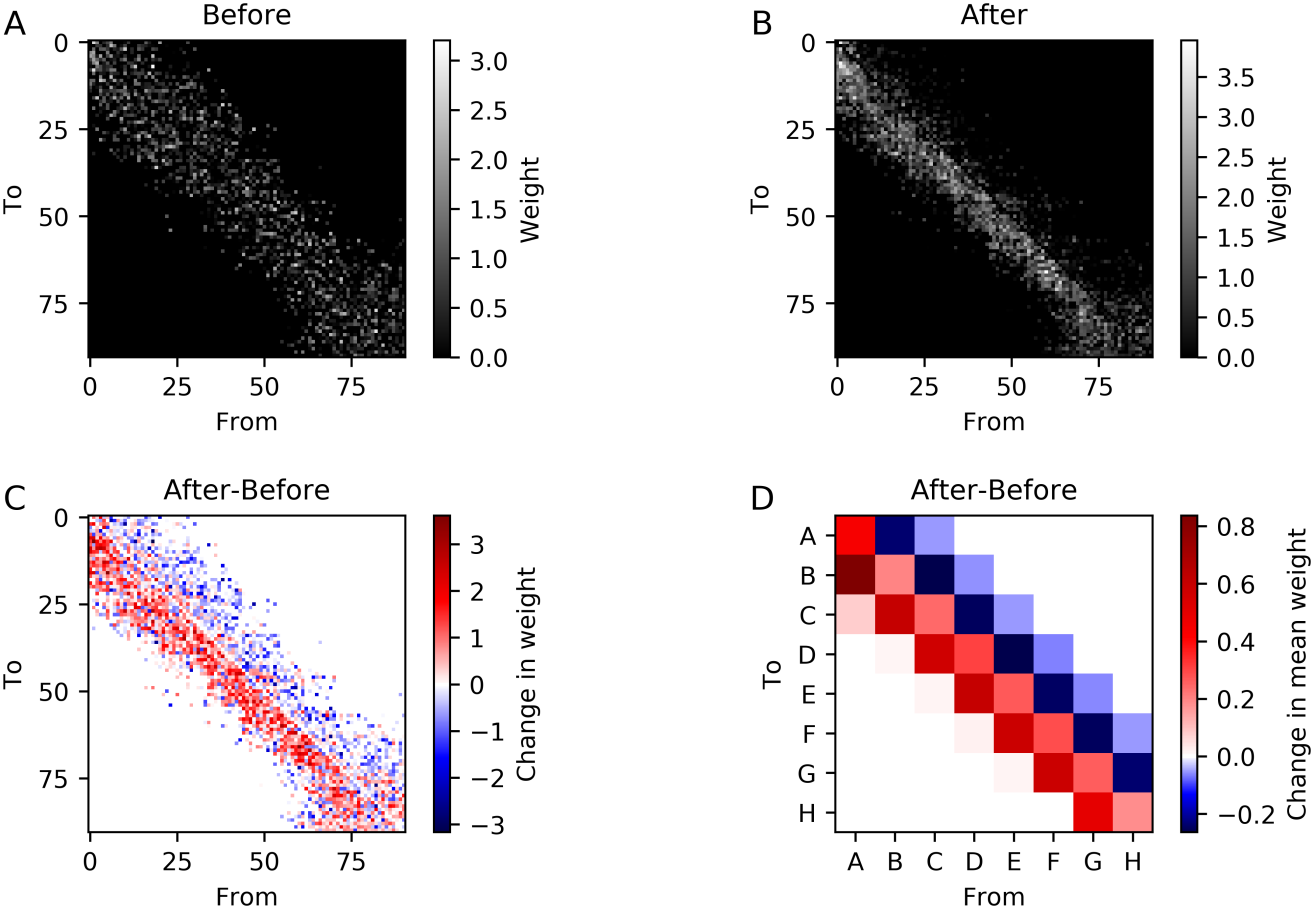
Effect of training with a moving spot along the $\tilde{S}\to \tilde{G}$ axis on connectivity. (A,B,C) Connection weights between neurons part of the clusters before (A) and after (B) training and their difference (C) for a single network instance. Neurons are ordered according to the projection of their position on the $\tilde{S}\to \tilde{G}$ axis. (D) Change of the mean weight of all possible connections between neurons part of the clusters. Averaged over 20 network instances.

We start by considering the weights between neurons part of one of the clusters *A*–*H* for one network instance. Fig 6A–6C show the connection weight matrix before and after training and their difference. Before training, we observed a structure with stronger weights distributed symmetrically around the diagonal. This reflected the distance dependency of the connectivity. After training, the symmetry was broken and the connections running in the direction of the moving spot used during training were strengthened while the connections in the opposite direction were weakened. To get a clearer picture of the weight change, we also determined the average connection weights between the different clusters *A*–*H* (Fig 6D). The connection weights between adjacent clusters in the forward direction were increasing due to training, while the opposite was true for the backward direction. The increase in connection weight was strongest for the *A* → *B* connections as these connections didn’t have as much SN-induced competition as the connections between adjacent clusters further along the $\tilde{S}\to \tilde{G}$ axis, since they had to compete with connections starting from other clusters located closer to $\tilde{S}$.

This stripe-like connectivity was a result of STDP and caused the increase in sequential spiking when triggering the sequence with a cue at $\tilde{S}$ and the decrease when triggering the sequence with a cue at $\tilde{G}$.

### Training affects spontaneous activity

Next, we examined if the training with a sequence had an impact on the spontaneous activity of the LIF-SORN. Therefor, we utilized the simulation protocol as described above with the difference that during testing no external input was used. Thus, only the noise drove the network during testing. As before, we determined the rate of each cluster by pooling the spikes of all neurons which were part of that cluster and convolving them with a Gaussian. However, we considered all spikes during the test phases and not only spikes within a window of 500 ms after stimulus presentation. Next we computed the times of all relative maxima of the firing rate for each cluster and ordered them. In the resulting sequence of firing times, we replaced each firing time with the corresponding cluster name *A*–*H*. Finally, we computed the transition probabilities between all clusters from this sequence. The transition probability from *A* to *D*, for example, was computed by dividing the number of times *D* was the cluster that fired directly after *A* by the total number of times *A* appeared. This was done for all combinations of clusters *A*–*H* and for the test phases before and after training.

**Fig 7:**
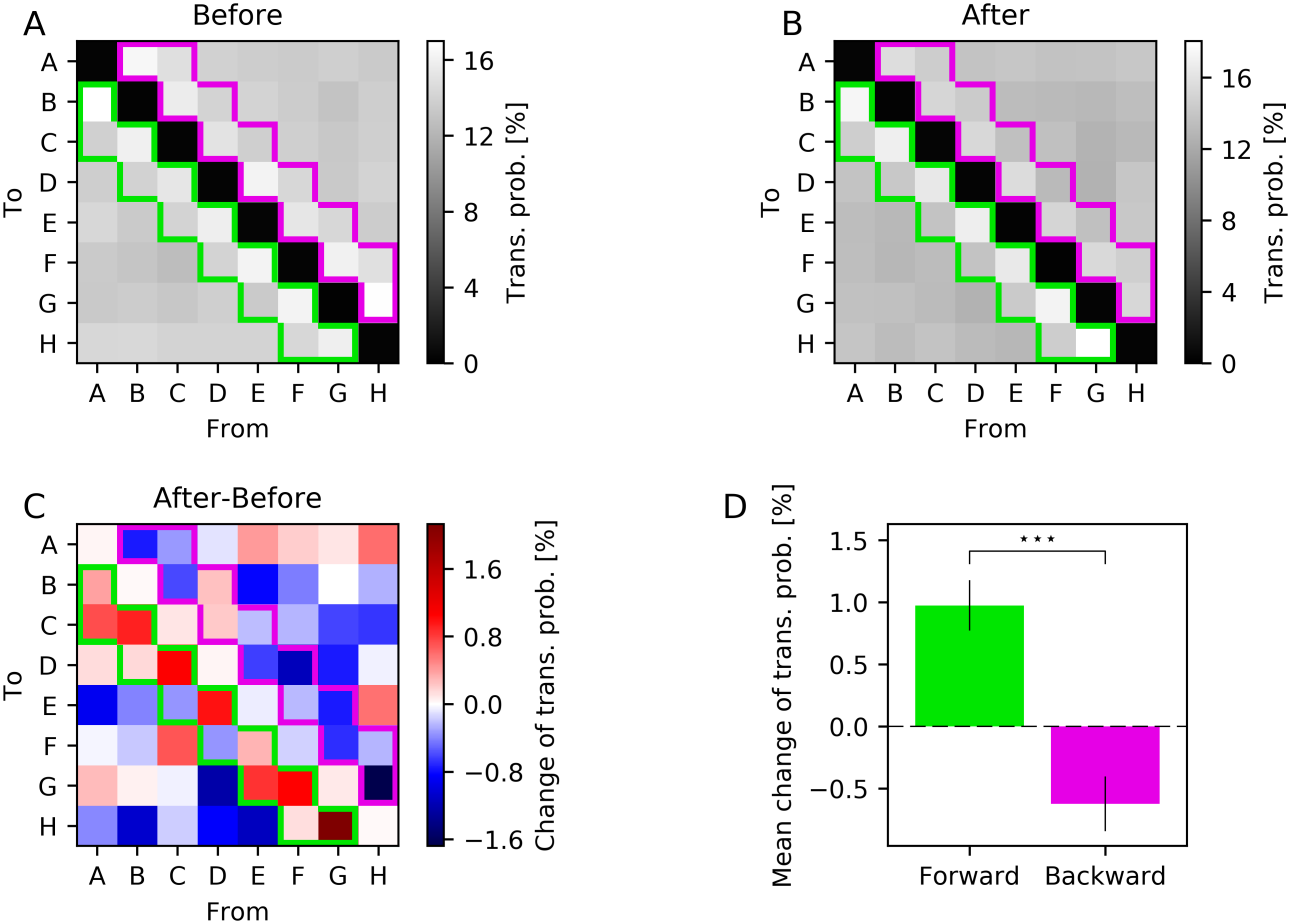
Effect of training with a moving spot along the $\tilde{S}\to \tilde{G}$ axis on spontaneous activity. (A,B,C) Transition probabilities between clusters before training (A), after training (B) and their difference (C). Colored frames indicate transitions in the forward (green) and backward (purple) direction. (D) Mean of the changes of transition probabilities in the forward (green) and backward (purple) direction corresponding to the framed transitions in (C). Errorbars show sem over network instances. Data from 30 network instances. Stars in panel D show significance (^⋆⋆⋆^ *P* < 0.001; Wilcoxon signed rank test).

Fig 7 shows the resulting transition probabilities before and after learning and their differences. Before and after learning, the transition probabilities from a cluster to itself were negligible and the transition probabilities between adjacent clusters as for example *A* to *B* or *D* to *C* were higher compared to the other transitions. This is not surprising as neurons close to one another strongly influenced each other due to the distance dependency of the connectivity. When considering the change in transition probabilities caused by the training, we observed that transitions between clusters in the $\tilde{S}\to \tilde{G}$ direction, which were separated by at most one other cluster, tended to be more likely while transitions between clusters in the opposite direction, which were separated by at most one other cluster, tended to be less likely. This finding was consistent with the weight change caused by the training (Fig 6D).

Thus, the characteristics of the sequence used during training were imprinted in the spontaneous activity of the LIF-SORN.

### Recall speed is independent of training speed

**Fig 8:**
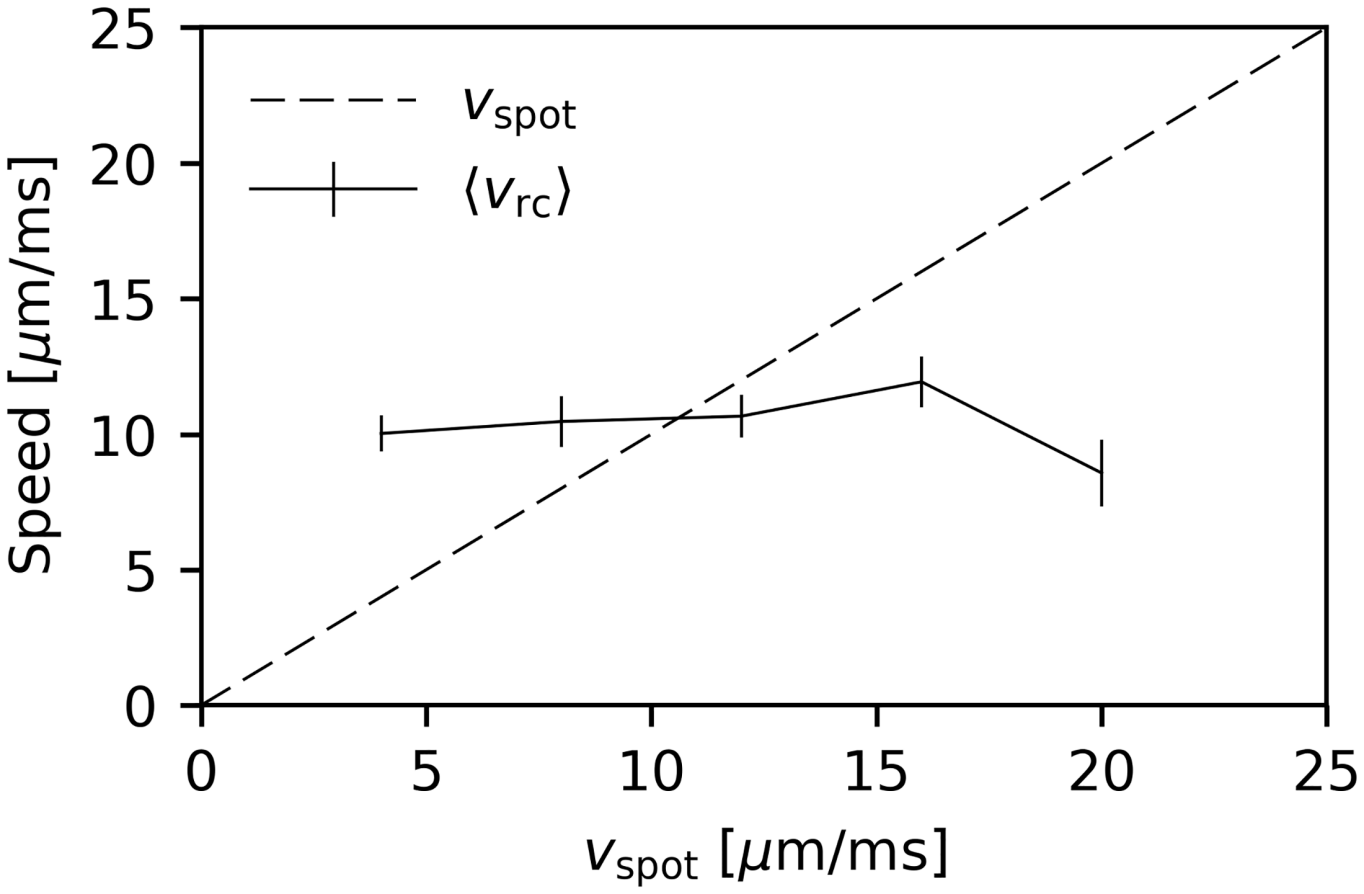
Mean recall speed as a function of the training speed. Mean recall speed of test trials with cue presentation at $\tilde{S}$ and a Spearman correlation coefficient greater than 0.9. Data from 20 network instances. Errorbars show sem.

So far, we were only concerned with the order of the replayed sequence and did not pay attention to its speed. In this section, we examine the recall speed *v*_rc_ after presentation of the test cue at $\tilde{S}$ with varying velocities *v*_spot_ of the motion sequence during training. We adopted the analysis of Xu et al. [1]. That is to say we considered only the test trials after learning for which the Spearman correlation coefficient was greater than 0.9. Then, we determined the recall speed for each of those trials by linear regression of the positions of the centers of each cluster as a function of their firing times. We found that the mean recall speed was independent of the training speed (Fig 8). It rather seemed to be determined by the network’s parameters.

Similar results were found by Xu et al. [1], i.e. they also observed no dependence of the recall speed on the speed used during training for anesthetized rats. Furthermore, spontaneous replay in cortex and hippocampus was found to be accelerated compared to training [29, 31]. All of these results suggest that only sequence order is learned and that the recall speed is primarily determined by the network’s dynamics and not the speed of the trained sequence. This observation on the level of local circuitry matches with the trivial fact that the recall of memories doesn’t happen with the speed with which they were experienced.

### Training effect is short-lasting

Xu et al. [1] also tested the persistence of the increase in sequential spiking caused by the training. To test this persistence in the LIF-SORN, we used a similar simulation protocol as before, i.e., training consisted of a moving spot shown along the $\tilde{S}\to \tilde{G}$ axis and testing of a briefly flashed spot at $\tilde{S}$. The duration of the test phase after training was tripled.

**Fig 9:**
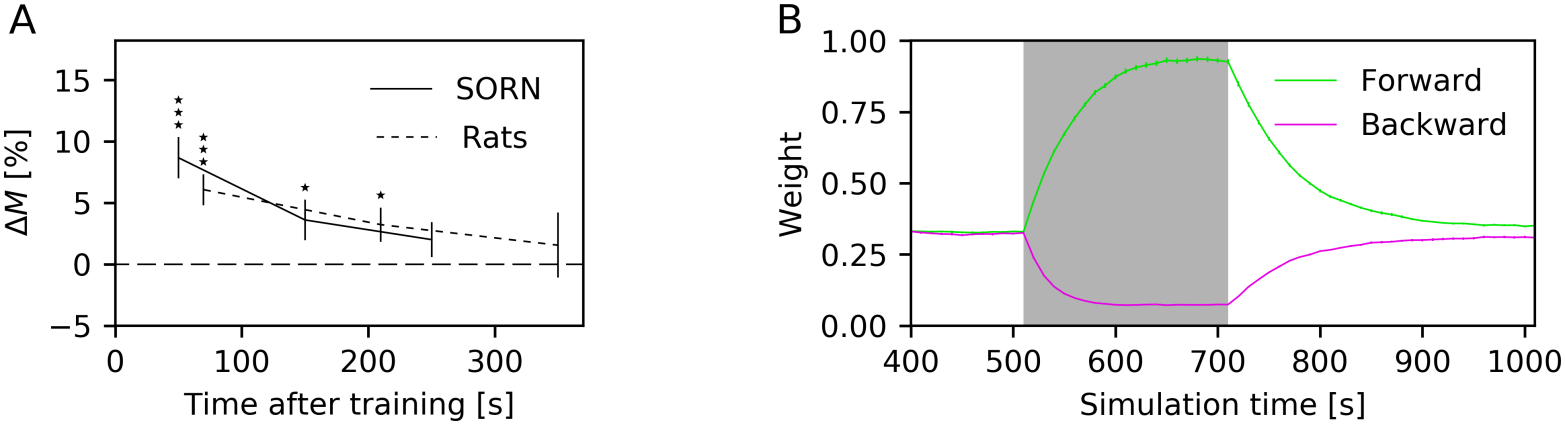
Persistence of training effect and time course of connection weights during and after training. (A) Change in percentage of matches as a function of time after training in the LIF-SORN and in rats [1]. (B) Time course of the connection weights between adjacent clusters in the forward (green) and backward (purple) direction before, during and after training. Shaded area marks the training phase. Data from 30 network instances. Dotted line in panel A shows approximate experimental data that were obtained from [1] using WebPlotDigitizer [30]. Errorbars show sem in all plots. Stars in panels A show significance (^⋆^ *P* < 0.05; ^⋆⋆^ *P* < 0.01; ^⋆⋆⋆^ *P* < 0.001; Wilcoxon signed rank test).

We adapted the analysis of Xu et al. [1] in that we defined a match as a test trial whose Spearman correlation coefficient was above a threshold of 0.6 and computed the change in percentage of matches during the test phase after training compared to the test phase before training. This was done for different times after training. We found that the effect of training as measured by the change in percentage of matches decayed within approximately 5 min (Fig 9A). The training effect decayed within a similar time, namely within around 7 min, in rats (Fig 9A). Hence, the training effect was short-lasting in both rats and the LIF-SORN.

Again, we can link the results obtained from the LIF-SORN’s activity with its connectivity. During training, the forward weights between adjacent clusters were increasing for approximately 100 s and then stayed roughly constant until the training ended as a consequence of the synaptic normalization. In the following test phase, the values of the weights from one cluster to the next were decaying back to their initial values, resulting in the simultaneous decay of the training effect on the network’s activity (Fig 9B). This decay was caused by the interplay of STDP with the asynchronous, irregular network activity.

## Discussion

Establishing the relationship between structure and function of cortical circuits remains a major challenge. Here we have presented a spiking neural network model of sequence learning in primary visual cortex that establishes a new conceptual bridge between structural and functional changes in cortical circuits. The model posits that STDP is the cellular basis for the sequence learning abilities of visual cortex. The temporally asymmetric shape of the STDP window (pre-before-post firing leads to potentiation, post-before-pre firing leads to depression) allows the circuit to detect the spatio-temporal structure of the stimulation sequence and lay it down in the circuit structure. The homeostatic mechanisms prevent runaway weight growth, among other functions. Importantly, while doing so the model also explains the origin of key structural features of the connectivity between the population of excitatory neurons. Among them are the lognormal-like distribution of synaptic efficacies, the distance-dependence of synaptic connection probabilities and the abundance of bidirectional connections between excitatory neurons.

There are many studies that have addressed the functioning of STDP in feed-forward models (e.g. [32, 33]). In addition, several previous studies have successfully modeled elements of sequence learning with STDP in recurrent networks [5, 6, 34–37], and another set of studies has attempted to account for the development of structural features of cortical wiring [20, 22, 38, 39]. However, our model is the first to combine both. Thereby it offers the most advanced unified account of the relation between structure and function of cortical circuits in the context of sequence learning. Furthermore, it does so from a self-organizing, bottom-up perspective, a critical component missing in most other examples of artificial sequence learning in recurrent neural networks [40–42].

Equally important, however, the model makes precise and testable predictions regarding how the excitatory-to-excitatory connectivity changes during learning. Specifically, it predicts that synaptic connections that project “in the direction of” the stimulation sequence are strengthened, while the reverse connections are weakened. Furthermore, it makes the testable prediction that spontaneous activity after learning should reflect the altered connectivity such that it leads to an increased probability of sequential activation. Similarly, replay of activity patterns is a well-known and widely studied phenomenon in hippocampus [43], but has also been observed in the neocortex [29, 31]. Despite these contributions, our model also has several limitations.

First, while our network reproduced most of the experimentally observed results by Xu et al. [1], namely enhanced sequential spiking in response to a cue at the start point of the sequence (Figs 4B and 5A), independence of the recall speed on the training speed (Fig 8) and a short persistence of the training effect (Fig 9A), it did not show the experimentally observed significant rightward shift of the Spearman correlation coefficient distribution in response to a cue at the midpoint (Fig 5B) and it exhibited an, experimentally not observed, small leftward shift of the Spearman correlation coefficient distribution in response to a cue at the goal point (Fig 5C).

Second, most of the chosen neuron and network parameters were taken from studies on layer 5 of rodent cortex [11, 12, 14], while Xu et al. [1] recorded from both deep and superficial layers.

Third, synaptic plasticity in our model was restricted to the connections among excitatory neurons. As a consequence, inhibition is unspecific in our network. From a functional perspective, this shouldn’t make much of a difference for the simple sequence learning task we considered. For more complex situations, such as multiple disparate assemblies, multiple sequences or branching sequences, this may be different, however. Adding plasticity mechanisms to the other connection types would also make the model more realistic and may allow it to establish additional links between the structure and function of cortical circuits in the context of sequence learning. This will be an interesting topic for future work.

Additional limitations exist in the model as a function of computational practicality. These include network size and related subsampling effects, as well as more complex input structures, noise correlations, etc. Overcoming these limitations would also be an interesting topic for future investigation.

Finally, in both the experiments of Xu et al. [1] and our model the training effect persists for only a short time. Xu et al. [1] astutely noted that even such short term storage can be quite useful for perceptual inference [44, 45], as repeated experiences in the recent past are often a good predictor of similar experiences in the near future. It is also clear, however, from perceptual learning experiments that visual cortex can store information for long periods of time [46]. So how are new memories protected from being quickly forgotten? How can they be stabilized for weeks, months, and years? This is an important question for future work.

## Methods

### Network model

The LIF-SORN is a recurrent neural network model of a small section of L5 of the rodent cortex. It consists of noisy leaky integrate-and-fire neurons and utilizes several biologically motivated plasticity mechanisms to self-organize its structure and activity. It was introduced by Miner and Triesch [22] with the plasticity mechanisms being short-term plasticity (STP), spike-timing dependent plasticity (STDP), synaptic normalization (SN), structural plasticity (SP), and intrinsic plasticity (IP). Here, we employed a modified version in comparison to this study. The most major changes were the use of a conductance based model instead of an additive model of synaptic transmission to make the synaptic signaling more realistic, the adjustment of the SN mechanism to account for boundary effects and the enlargement of the network to a size that is similar to size of the cortical region considered by Xu et al. [1].

The parameter values in the LIF-SORN are set in accordance to experimental data from L5 of the cortex, although the timescales of SN, SP and IP are accelerated compared to biological findings in order to decrease the necessary simulation time. See [22] for a more detailed explanation of the selection of the individual values than the explanations given below. The network was simulated with the help of the Brian spiking neural network simulator [47] using a simulation timestep of Δ*t*_sim_ = 0.1 ms.

#### Network architecture

The LIF-SORN as used in this study consists of *N*^E^ = 1000 excitatory and *N*^I^ = 0.2 × *N*^E^ = 200 inhibitory neurons, which are placed randomly on a 2500 μm × 1000 μm grid (Fig 1A). The connectivity between them is distance-dependent in approximate accordance to experimental data [12, 14], i.e., all possible connections are assigned a probability according to their length, determined from a Gaussian probability function with a mean of 0 μm and a half width of 200 μm. A connection fraction of 0.1 of possible synapses between excitatory and inhibitory neurons and vice versa are realized as well as a connection fraction of 0.5 of reciprocal potential connections between inhibitory neurons corresponding to experimentally observed values from L5 of the rodent cortex [11]. Connections among excitatory neurons are initially not realized, but are created during a growth phase as a consequence of SP. Those recurrent excitatory synapses are the only type of connections subject to plasticity.

#### Neuron and synapse model

The subthreshold dynamics of the membrane potential *V*_*n*_ of a neuron *n*, which could be either excitatory or inhibitory, is governed by

$$\begin{array}{ll} \frac{\text{d}V_{n}}{\text{d}t}\left(t\right)= & -\frac{V_{n}\left(t\right)-E_{\text{L}}}{\tau }\\ & -\frac{\left(g_{\text{e},n}\left(t\right)+g_{\text{ext},n}\left(t\right)\right)\left(V_{n}\left(t\right)-E_{\text{e}}\right)}{\tau }\\ & -\frac{g_{\text{i},n}\left(t\right)\left(V_{n}\left(t\right)-E_{\text{i}}\right)}{\tau }+\frac{\sigma \xi \left(t\right)}{\sqrt{\tau }}, \end{array}$$ (2)

where *E*_L_ = −60 mV is the resting potential, *τ* = 20 ms is the membrane time constant, *ξ*(*t*) is Gaussian white noise and *σ* = 16 mV is the standard deviation of the noise. *E*_e_ = 0 mV is the reversal potential for connections starting at excitatory neurons and *g*_e,*n*_(*t*) is a dimensionless measure for their synaptic conductance. It is determined by

$$\frac{\text{d}g_{\text{e},n}}{\text{d}t}\left(t\right)=-\frac{g_{\text{e},n}\left(t\right)}{\tau_{\text{e}}}+\sum_{m_{\text{e}}}W_{m_{\text{e}}n}^{\text{eff}}\left(t\right)\sum_{f_{m_{\text{e}}}}\delta \left(t-t_{f_{m_{\text{e}}}}-t_{m_{\text{e}}n}\right),$$ (3)

where *τ*_*e*_ = 3 ms is the synaptic time constant for excitatory connections, *m*_e_ indexes the excitatory neurons, $W_{m_{\text{e}}n}^{\text{eff}}\left(t\right)$ is the dimensionless effective connection weight between neuron *m*_e_ and *n*, $t_{m_{\text{e}}n}$ is the conduction delay between neuron *m*_e_ and *n* and $f_{m_{\text{e}}}$ indexes the spike times of neuron *m*_e_. *g*_ext,*n*_(*t*) describes potential synaptic input originating from outside the network (see below). Similarly, *E*_i_ = −80 mV is the reversal potential for connections starting at inhibitory neurons and *g*_i,*n*_(*t*) is a dimensionless measure for their synaptic conductance. Its time evolution is governed by

$$\frac{\text{d}g_{\text{i},n}}{\text{d}t}\left(t\right)=-\frac{g_{\text{i},n}\left(t\right)}{\tau_{\text{i}}}+\sum_{m_{\text{i}}}W_{m_{\text{i}}n}\sum_{f_{m_{\text{i}}}}\delta \left(t-t_{f_{m_{\text{i}}}}-t_{m_{\text{i}}n}\right),$$ (4)

where *τ*_*i*_ = 5 ms is the synaptic time constant for inhibitory connections, *m*_i_ indexes the inhibitory neurons, *W*_*m*_i_*n*_ is the connection weight between neuron *m*_i_ and *n*, $t_{m_{\text{i}}n}$ is the conduction delay between neuron *m*_i_ and *n* and $f_{m_{\text{i}}}$ indexes the spike times of neuron *m*_i_.

If the membrane potential rises above a threshold potential, the model neuron fires a spike. Afterwards, the membrane potential is returned to a reset potential. For excitatory neurons, the threshold potential $V_{\text{T}}^{\text{E}}\left(t\right)$ is variable due to intrinsic plasticity and the reset potential is given by $V_{\text{reset}}^{\text{E}}=-70\;\text{mV}$. For inhibitory neurons, the threshold potential reads $V_{\text{T}}^{\text{I}}=-48\;\text{mV}$ and the reset potential is $V_{\text{reset}}^{\text{I}}=-60\;\text{mV}$.

The connections starting from inhibitory neurons are initially inserted with a weight of 0.4 and a conduction delay of 2 ms, the connections between excitatory and inhibitory units are inserted with a weight of 0.15 and exhibit a conduction delay of 1 ms and the conduction delay between excitatory neurons is 3 ms while the connection strength is variable because of the plasticity mechanisms. All conduction delays are assumed to be homogeneous and purely axonal.

#### Plasticity mechanisms

Short term plasticity (STP) modulates the weight $W_{m_{\text{e}}n_{\text{e}}}\left(t\right)$ of the connection between excitatory neurons *m*_e_ and *n*_e_ on a short time scale based on the firing history of the presynaptic neuron *m*_e_ [48]. Specifically, short term facilitation is described by a variable $u_{m_{\text{e}}}\left(t\right)$ and short term depression by a variable $x_{m_{\text{e}}}\left(t\right)$. Their dynamics are governed by

$$\frac{\text{d}u_{m_{e}}}{\text{d}t}\left(t\right)=\frac{U-u_{m_{\text{e}}}\left(t\right)}{\tau_{\text{f}}}+U\left(1-u_{m_{\text{e}}}\left(t^{-}\right)\right)\underset{f_{m_{\text{e}}}}{\sum }\delta \left(t-t_{f_{m_{\text{e}}}}-t_{\text{ee}}\right),$$ (5)

$$\frac{\text{d}x_{m_{e}}}{\text{d}t}\left(t\right)=\frac{1-x_{m_{\text{e}}}\left(t\right)}{\tau_{\text{d}}}+x_{m_{\text{e}}}\left(t^{-}\right)u_{m_{\text{e}}}\left(t^{-}\right)\underset{f_{m_{\text{e}}}}{\sum }\delta \left(t-t_{f_{m_{\text{e}}}}-t_{\text{ee}}\right),$$ (6)

where *t*_ee_ = 3 ms is the conduction delay between excitatory neurons, *U* = 0.04 is the increment of $u_{m_{\text{e}}}\left(t\right)$ produced by a presynaptic spike and *τ*_*d*_ = 500 ms and *τ*_*f*_ = 2000 ms are the respective depression and facilitation timescales. The parameter values approximate the experimentally observed values from [48] and [49]. $f_{m_{\text{e}}}$ indexes the presynaptic spikes and *t*^−^ indicates the point in time right before spike arrival at the synapse. The effective connection weight at spike arrival is then given by $W_{m_{\text{e}}n_{\text{e}}}^{\text{eff}}\left(t\right)=W_{m_{\text{e}}n_{\text{e}}}\left(t\right)\times u_{m_{\text{e}}}\left(t^{-}\right)\times x_{m_{\text{e}}}\left(t^{-}\right)$. STP increases network stability in that it leads to a larger parameter regime in which the network exhibits asynchronous irregular firing.

Exponential spike-timing dependent plasticity (STDP) changes the weight $W_{m_{\text{e}}n_{\text{e}}}\left(t\right)$ of the connection between excitatory neurons *m*_e_ and *n*_e_ by adding

$$\begin{array}{c} \Delta W_{m_{\text{e}}n_{\text{e}}}=\sum_{f_{m_{\text{e}}}}\sum_{j_{n_{\text{e}}}}W\left(t_{j_{n_{\text{e}}}}-t_{f_{m_{\text{e}}}}-t_{\text{ee}}\right) \end{array}$$ (7)

to it upon a spike of one of the neurons, wherein $W_{m_{\text{e}}n_{\text{e}}}\left(t\right)$ has a lower bound of 0 mV. Here, $f_{m_{\text{e}}}$ indexes the presynaptic and $j_{n_{\text{e}}}$ the postsynaptic spikes. *t*_ee_ = 3 ms is the conduction delay between excitatory neurons. The weight change is determined by an asymmetric STDP window that reads

$$\begin{array}{c} W\left(\Delta t\right)=\{\begin{array}{cc} A_{+}\exp\left(-\frac{\Delta t}{\tau_{+}}\right)\; & \text{if}\;\;\;\Delta t>0\;,\\ A_{-}\exp\left(\frac{\Delta t}{\tau_{-}}\right)\; & \text{if}\;\;\;\Delta t<0\;,\\ 0\; & \text{if}\;\;\;\Delta t=0\;, \end{array} \end{array}$$ (8)

where *A*_+_ = 4.8 × 10^−2^ and *A*_−_ = −2.4 × 10^−2^ are the amplitudes and *τ*_+_ = 15 ms and *τ*_−_ = 30 ms the time constants of the weight change in rough accordance to [2] and [50]. To save simulation time, only the nearest neighbors of pre- and postsynaptic spikes are considered [51].

STDP endows the network with the ability to learn correlations in external input. However, when using recurrent neural networks that solely include STDP, one encounters problems arising from a positive feedback loop: a change in the connectivity modifies the network activity which again modulates the connectivity and so on. This feedback process happens in an uncontrolled fashion and can lead to unbounded growth of synaptic strengths, which destabilizes the network. Furthermore, as STDP is changing the connection weights individually, the neurons are potentially loosing their selectivity for different synaptic inputs. Thus the ability of those neural networks to process information is highly suppressed. To cope with this problem, the LIF-SORN is endowed with homeostatic plasticity mechanisms [16–19].

Synaptic normalization (SN) is one of the homeostatic plasticity mechanisms used in our model. It scales the total synaptic drive the neurons receive. SN is implemented by updating all recurrent excitatory weights according to the rule

$$\begin{array}{c} W_{m_{\text{e}}n_{\text{e}}}\left(t\right)\to W_{\text{total}}\left(n_{\text{e}}\right)\frac{W_{m_{\text{e}}n_{\text{e}}}\left(t\right)}{\sum_{m_{\text{e}}}W_{m_{\text{e}}n_{\text{e}}}} \end{array}$$ (9)

once per second. Here, *W*_total_(*n*_e_) is the target total input for neuron *n*_e_. It is calculated by multiplying the target connection fraction by the size of the incoming neuron population, the mean synapse strength, which is chosen to be 0.8 for the recurrent excitatory synapses, and the factor

$$\int_{0\;\mu \text{m}}^{2500\;\mu \text{m}}\text{d}x\int_{0\;\mu \text{m}}^{1000\;\mu \text{m}}\text{d}y\frac{1}{2\pi \sigma^{2}}\text{exp}\left(\frac{∥x-x_{n_{\text{e}}}{∥}^{2}}{2\sigma^{2}}\right),$$ (10)

where $x_{n_{\text{e}}}$ is the position of neuron *n*_e_ and *σ* = 200 μm is the half width of the Gaussian probability function used to assign a distance-dependent probability to each possible connection. This factor accounts for the fact that neurons close to the network boundaries form less connections than neurons in the middle of the network. Hence, the mean weights of the connections projecting to neurons close to the boundaries are, without this factor, higher than the mean weights of the neurons in the middle of the network. This leads to spontaneous activity waves that start at the corners of the network and subsequently lead to the firing of all other network neurons. To avoid these waves, we introduced the aforementioned factor, which is simply the integral of a normal distribution centered at the neuron’s position over the network dimension. Furthermore, the other connection types are normalized in the same way once before the simulation starts.

The SN mechanism of our network is motivated by the phenomenon observed in [52], in which it was shown that, during long-term potentiation, the overall density of postsynaptic AMPA receptors per micrometer of dendrite is approximately preserved, while the density at some synapses increases.

In addition to STDP and SN, which modify the weights of existing connections, a form of structural plasticity (SP) that implements growth and pruning of recurrent excitatory connections is included in the LIF-SORN. Synaptic growth is modeled by adding once per second a random number of connections with an initial weight of 1 × 10^−3^ to the network. The number of new synapses is drawn from a Gaussian distribution with a mean of 6000 connections per second and a standard deviation of $\sqrt{6000}$ connections per second in order to achieve a connection fraction of approximately 0.1 [11, 12]. The specific connections that are to be added to the network are selected according to the distance-dependent probability assigned to each connection. As described above, this connection probability is calculated from a Gaussian probability function with a mean of 0 μm and a standard deviation of 200 μm. Synaptic pruning is implemented by eliminating all connections whose weight is below a threshold of 1 × 10^−4^ once per second.

In addition to the synaptic plasticity mechanisms and the structural plasticity, an intrinsic plasticity (IP) mechanism that regulates the firing threshold $V_{\text{T},n_{\text{e}}}\left(t\right)$ for each excitatory neuron *n*_e_ is used. IP is implemented by updating the threshold at every simulation timestep according to the rule

$$\begin{array}{c} V_{\text{T},n_{\text{e}}}\left(t\right)\to V_{\text{T},n_{\text{e}}}\left(t\right)+\eta_{\text{IP}}(N_{\text{spikes}}-h_{\text{IP}})\;, \end{array}$$ (11)

where *N*_spikes_ = 1 if the neuron has spiked in the previous timestep and *N*_spikes_ = 0 otherwise, *η*_IP_ = 0.1 mV is the learning rate and *h*_IP_ = *r*_target_ × Δ*t*_sim_ = 3 × 10^−4^ is the target number of spikes per update interval with *r*_target_ = 3 Hz being the target firing rate. IP is a homeostatic mechanism that stabilizes network activity at the level of individual neurons. Note that this simple IP mechanism assigns the same target firing rate to each neuron. A more complex diffusive IP mechanism has recently been shown to produce a broad distribution of firing rates [53] as observed in the cortex. The inclusion of such a mechanism is currently being worked on.

While it is, at least to our knowledge, not possible to pinpoint a single biological mechanism that matches the IP in our network, a couple of mechanisms that affect the intrinsic excitability of neurons have been observed. One of these mechanisms is spike-rate adaptation, which quickly reduces neuronal firing in response to continuous drive [54]. Other observed mechanisms modify intrinsic excitability on slower timescales [55, 56].

### Modeling of sequence learning

#### Summary of experimental set-up used by Xu et al. [1]

We tried to match our simulation of sequence learning closely to the experimental set-up and stimulation paradigm of the study by Xu et al. [1]. Their experimental set-up comprised head-fixed rats with a multielectrode array inserted in their primary visual cortex and a LCD-screen placed in front of their left eye. Both awake and anesthetized rats were investigated, but since the LIF-SORN acts in a regime of asynchronous irregular spiking, we focused on their results from awake rats. The multielectrode array consisted of 2 × 8 channels with 250 μm between neighboring channels and was inserted in the right primary visual cortex. Before the actual experiments, the receptive field of each channel was determined. In most experiments, the visual stimulus was a bright light spot, which was moved during conditioning along the long axis of the distribution of receptive fields from a start point $\tilde{S}$ to an end, or goal, point $\tilde{G}$ over a period of 400 ms–600 ms. The moving spot evoked sequential responses recorded by the multielectrode array. To examine the effect of the conditioning, the responses to different cues, as for example a brief flash of the light spot at $\tilde{S}$, were recorded before and after conditioning.

#### Modeling input in LIF-SORN

We modeled the movement of the light stimulus and its influence on the network by sweeping a spot that drives the excitatory neurons across the network. Assuming that the stimulus is presented to the network in an interval *I*_spot_ = [*t*_0_, *t*_0_ + *t*_spot_], the trajectory of the spot is given by

$$\begin{array}{c} u\left(t\right)=x_{\tilde{\text{S}}}+\left(x_{\tilde{\text{G}}}-x_{\tilde{\text{S}}}\right)\frac{t-t_{0}}{t_{\text{spot}}}\;, \end{array}$$ (12)

where $x_{\tilde{\text{S}}}={\left(375\;\mu \text{m},\;500\;\mu \text{m}\right)}^{\text{T}}$ is the start and $x_{\tilde{\text{G}}}={\left(2125\;\mu \text{m},\;500\;\mu \text{m}\right)}^{\text{T}}$ the end point of the movement (Fig 1C). The spot reflected the activity of input spike trains, i.e., an excitatory neuron *n*_e_ located at position $x_{n_{\text{e}}}$ received *N*_input_ Poissonian spike trains with rate

$$\begin{array}{c} r_{\text{spot}}\left(x_{n_{\text{e}}},t\right)=\{\begin{array}{ll} r_{\text{max}}\text{exp}\left(-{\left(\frac{∥x_{n_{\text{e}}}-u\left(t\right)∥}{\alpha }\right)}^{\beta }\right) & \text{if}t\in I_{\text{spot}},\\ 0\;\text{Hz} & \text{else}, \end{array} \end{array}$$

where *r*_max_ is the maximal rate of the spike trains, *α* determines the scale and *β* the shape of the spot. These spike trains drive the excitatory neurons via extra input conductances. For neuron *n*_e_, the time evolution of this extra conductance is governed by

$$\begin{array}{c} \frac{\text{d}g_{\text{ext},n_{\text{e}}}}{\text{d}}\left(t\right)=-\frac{g_{\text{ext},n_{\text{e}}}\left(t\right)}{\tau_{\text{e}}}+\sum_{l=1}^{N_{\text{input}}}W_{\text{FF}}\sum_{k_{l}}\delta \left(t-t_{k_{l}}\right), \end{array}$$ (13)

where *W*_FF_ is the weight of the feedforward input and the $t_{k_{l}}$ are the spike times of the Poissonian input spike trains.

The values of the parameters were set to achieve similar activity as in [1]. Unless noted otherwise, we used *r*_max_ = 50 Hz, *N*_input_ = 100, *W*_FF_ = 0.04, *α* = 150 μm, *β* = 4 and $t_{\text{spot}}=\frac{∥x_{\tilde{\text{G}}}-x_{\tilde{\text{S}}}∥}{v_{\text{spot}}}$ with *v*_spot_ = 4 μm/ms. Fig 1B shows the cross-section of *r*_spot_(***x***, *t*). To model the presentation of a cue as for example a brief flash of the light spot at the starting point, we used *t*_spot_ = 100 ms and kept ***u***(*t*) fixed to one position (e.g. $x_{\tilde{\text{S}}}$) independent of time.

#### Modeling recording in LIF-SORN

For the analysis of the sequence learning, we defined *n*_clu_ = 8 clusters of excitatory neurons that were encompassed by circles with a radius of *r*_clu_ = 100 μm. Their centers were placed equidistantly on the line between $x_{\tilde{\text{S}}}$ and $x_{\tilde{\text{G}}}$ with the boundaries of the outermost circles touching $x_{\tilde{\text{S}}}$ and $x_{\tilde{\text{G}}}$, respectively (Fig 1C). We then defined the activity of each cluster to comprise the spikes of the neurons of the cluster. For the sake of readability, we named the clusters *A* to *H* according to their distance to $x_{\tilde{\text{S}}}$. These clusters and their parameters were chosen to achieve a middle ground between two goals. The first goal was the reproduction of the recording with a multi-electrode array, where one electrode is actually only recording the activity of few neurons close to the electrode. The second goal was the inclusion of enough neurons per cluster to obtain a number of spikes sufficient to get meaningful results since the spiking of the neurons was quite variable.

## Supporting information

S1 Appendix Conversion of weights to PSP amplitudes. (PDF)

S1 FigCumulative distribution of Spearman correlation coefficients for different training paradigms.(A,B) Results for training with a moving spot whose trajectory is shifted in a direction orthogonal to the $\tilde{S}\to \tilde{G}$ axis. In the LIF-SORN, the clusters were aligned between $x_{\tilde{\text{S}}}={\left(375\;\mu \text{m},\;350\;\mu \text{m}\right)}^{\text{T}}$ and $x_{\tilde{\text{G}}}={\left(2125\;\mu \text{m},\;350\;\mu \text{m}\right)}^{\text{T}}$, while the spot moved from (375 μm, 650 μm)^T^ to (2125 μm, 650 μm)^T^ during training. (C,D) Results for training by flashing a bar that spans from $\tilde{S}$ to $\tilde{G}$. (E,F) Results for training by flashing a spot at $\tilde{S}$. Plots showing results of the LIF-SORN are based on data from 10 network instances. Plots showing results of rats were obtained from awake (bar-stimulus) and anesthetized (parallel shifted sequence, flash-stimulus) rats and were extracted from [1] using WebPlotDigitizer [30].(TIF)

## References

[1] Xu S, Jiang W, Poo MM, Dan Y. Activity recall in a visual cortical ensemble. Nat Neurosci. 2012;15(3):449–455. doi: 10.1038/nn.3036 22267160 10.1038/nn.3036 PMC3288189

[2] Bi GQ, Poo MM. Synaptic modifications in cultured hippocampal neurons: dependence on spike timing, synaptic strength, and postsynaptic cell type. J Neurosci. 1998;18(24):10464–10472. doi: 10.1523/JNEUROSCI.18-24-10464.1998 9852584 10.1523/JNEUROSCI.18-24-10464.1998 PMC6793365

[3] Song S, Miller KD, Abbott LF. Competitive Hebbian learning through spike-timing-dependent synaptic plasticity. Nat Neurosci. 2000;3(9):919–926. doi: 10.1038/78829 10966623 10.1038/78829

[4] Byrnes S, Burkitt AN, Grayden DB, Meffin H. Learning a Sparse Code for Temporal Sequences Using STDP and Sequence Compression. Neural Comput. 2011;23(10):2567–2598. doi: 10.1162/NECO_a_00184 21732857 10.1162/NECO_a_00184

[5] Fiete IR, Senn W, Wang CZH, Hahnloser RHR. Spike-Time-Dependent Plasticity and Heterosynaptic Competition Organize Networks to Produce Long Scale-Free Sequences of Neural Activity. Neuron. 2010;65(4):563–576. doi: 10.1016/j.neuron.2010.02.003 20188660 10.1016/j.neuron.2010.02.003

[6] Lazar A, Pipa G, Triesch J. SORN: a Self-organizing Recurrent Neural Network. Front Comput Neurosci. 2009;3:1–9. doi: 10.3389/neuro.10.023.2009 19893759 10.3389/neuro.10.023.2009 PMC2773171

[7] Masquelier T, Guyonneau R, Thorpe SJ. Competitive STDP-Based Spike Pattern Learning. Neural Comput. 2009;21(5):1259–1276. doi: 10.1162/neco.2008.06-08-804 19718815 10.1162/neco.2008.06-08-804

[8] Toutounji H, Pipa G. Spatiotemporal Computations of an Excitable and Plastic Brain: Neuronal Plasticity Leads to Noise-Robust and Noise-Constructive Computations. PLoS Comput Biol. 2014;10(3):e1003512 doi: 10.1371/journal.pcbi.1003512 24651447 10.1371/journal.pcbi.1003512 PMC3961183

[9] Harris KM, Stevens JK. Dendritic spines of CA 1 pyramidal cells in the rat hippocampus: serial electron microscopy with reference to their biophysical characteristics. J Neurosci. 1989;9(8):2982–2997. doi: 10.1523/JNEUROSCI.09-08-02982.1989 2769375 10.1523/JNEUROSCI.09-08-02982.1989 PMC6569708

[10] Lisman JE, Harris KM. Quantal analysis and synaptic anatomy—integrating two views of hippocampal plasticity. Trends Neurosci. 1993;16(4):141–147. doi: 10.1016/0166-2236(93)90122-3 7682347 10.1016/0166-2236(93)90122-3

[11] Thomson AM, West DC, Wang Y, Bannister AP, Free R, Street RH, et al Synaptic Connections and Small Circuits Involving Excitatory and Inhibitory Neurons in Layers 2–5 of Adult Rat and Cat Neocortex: Triple Intracellular Recordings and Biocytin Labelling In Vitro. Cereb Cortex. 2002;12:936–953. doi: 10.1093/cercor/12.9.936 12183393 10.1093/cercor/12.9.936

[12] Song S, Sjöström PJ, Reigl M, Nelson S, Chklovskii DB. Highly Nonrandom Features of Synaptic Connectivity in Local Cortical Circuits. PLoS Biol. 2005;3(3):e68 doi: 10.1371/journal.pbio.0030068 15737062 10.1371/journal.pbio.0030068 PMC1054880

[13] Yasumatsu N, Matsuzaki M, Miyazaki T, Noguchi J, Kasai H. Principles of Long-Term Dynamics of Dendritic Spines. J Neurosci. 2008;28(50):13592–13608. doi: 10.1523/JNEUROSCI.0603-08.2008 19074033 10.1523/JNEUROSCI.0603-08.2008 PMC2706274

[14] Perin R, Berger TK, Markram H. A synaptic organizing principle for cortical neuronal groups. Proc Natl Acad Sci. 2011;108(13):5419–5424. doi: 10.1073/pnas.1016051108 21383177 10.1073/pnas.1016051108 PMC3069183

[15] Markram H. A network of tufted layer 5 pyramidal neurons. Cereb Cortex. 1997;7(6):523–533. doi: 10.1093/cercor/7.6.523 9276177 10.1093/cercor/7.6.523

[16] Abbott LF, Nelson SB. Synaptic plasticity: taming the beast. Nat Neurosci. 2000;3:1178–1183. doi: 10.1038/81453 11127835 10.1038/81453

[17] Turrigiano GG, Nelson SB. Homeostatic plasticity in the developing nervous system. Nat Rev Neurosci. 2004;5(2):97–107. doi: 10.1038/nrn1327 14735113 10.1038/nrn1327

[18] Turrigiano GG. Homeostatic Synaptic Plasticity: Local and Global Mechanisms for Stabilizing Neuronal Function. Cold Spring Harb Perspect Biol. 2012;4(1):a005736 doi: 10.1101/cshperspect.a005736 22086977 10.1101/cshperspect.a005736 PMC3249629

[19] Vitureira N, Goda Y. The interplay between hebbian and homeostatic synaptic plasticity. J Cell Biol. 2013;203(2):175–186. doi: 10.1083/jcb.201306030 24165934 10.1083/jcb.201306030 PMC3812972

[20] Zheng P, Dimitrakakis C, Triesch J. Network Self-Organization Explains the Statistics and Dynamics of Synaptic Connection Strengths in Cortex. PLoS Comput Biol. 2013;9(1):e1002848 doi: 10.1371/journal.pcbi.1002848 23300431 10.1371/journal.pcbi.1002848 PMC3536614

[21] Duarte R, Seriès P, Morrison A. Self-Organized Artificial Grammar Learning in Spiking Neural Networks. In: Proc. 36th Annu. Conf. Cogn. Sci. Soc.; 2014. p. 427–432.

[22] Miner D, Triesch J. Plasticity-Driven Self-Organization under Topological Constraints Accounts for Non-random Features of Cortical Synaptic Wiring. PLoS Comput Biol. 2016;12(2):e1004759 doi: 10.1371/journal.pcbi.1004759 26866369 10.1371/journal.pcbi.1004759 PMC4750861

[23] Wang Q, Rothkopf CA, Triesch J. A model of human motor sequence learning explains facilitation and interference effects based on spike-timing dependent plasticity. PLoS Comput Biol. 2017;13(8):e1005632 doi: 10.1371/journal.pcbi.1005632 28767646 10.1371/journal.pcbi.1005632 PMC5555713

[24] Vaadia E, Aertsen A. In: Aertsen A, Braitenberg V, editors. Coding and Computation in the Cortex: Single-Neuron Activity and Cooperative Phenomena. Berlin: Springer; 1992 p. 81–121.

[25] Abeles M. Corticonics: Neural Circuits of the Cerebral Cortex. Cambridge: Cambridge University Press; 1991.

[26] Duarte R, Morrison A. Dynamic stability of sequential stimulus representations in adapting neuronal networks. Front Comput Neurosci. 2014;8:124 doi: 10.3389/fncom.2014.00124 25374534 10.3389/fncom.2014.00124 PMC4205815

[27] Abbott LF, Dayan P. Theoretical Neuroscience. Cambridge: MIT Press; 2001.

[28] Mason A, Nicoll A, Stratford K. Synaptic Transmission between Individual Pyramidal Neurons of the Rat Visual Cortex in vitro. J Neurosci. 1991;11(1):72–84. doi: 10.1523/JNEUROSCI.11-01-00072.1991 1846012 10.1523/JNEUROSCI.11-01-00072.1991 PMC6575189

[29] Euston DR, Tatsuno M, McNaughton BL. Fast-Forward Playback of Recent Memory Sequences in Prefrontal Cortex During Sleep. Science. 2007;318(5853):1147–1150. doi: 10.1126/science.1148979 18006749 10.1126/science.1148979

[30] Rohatgi A. WebPlotDigitizer v3.12; 2017. Available from: http://arohatgi.info/WebPlotDigitizer.

[31] Ji D, Wilson MA. Coordinated memory replay in the visual cortex and hippocampus during sleep. Nat Neurosci. 2007;10(1):100–107. doi: 10.1038/nn1825 17173043 10.1038/nn1825

[32] Mehta MR, Wilson MA. From hippocampus to V1: Effect of LTP on spatio-temporal dynamics of receptive fields. Neurocomputing 2000;32:905–911. doi: 10.1016/S0925-2312(00)00259-9

[33] Mehta MR, Quirk MC, Wilson MA. Experience-Dependent Asymmetric Shape of Hippocampal Receptive Fields. Neuron 2000;25(3):707–715. doi: 10.1016/S0896-6273(00)81072-7 10774737 10.1016/s0896-6273(00)81072-7

[34] Liu JK, Buonomano DV. Embedding Multiple Trajectories in Simulated Recurrent Neural Networks in a Self-Organizing Manner. J Neurosci. 2009;29(42):13172–13181. doi: 10.1523/JNEUROSCI.2358-09.2009 19846705 10.1523/JNEUROSCI.2358-09.2009 PMC6665184

[35] Kappel D, Nessler B, Maass W. STDP Installs in Winner-Take-All Circuits an Online Approximation to Hidden Markov Model Learning. PLoS Comput Biol. 2014;10(3):e1003511 doi: 10.1371/journal.pcbi.1003511 24675787 10.1371/journal.pcbi.1003511 PMC3967926

[36] Jahnke S, Timme M, Memmesheimer RM. A Unified Dynamic Model for Learning, Replay, and Sharp-Wave/Ripples. J Neurosci. 2015;35(49):16236–16258. doi: 10.1523/JNEUROSCI.3977-14.2015 26658873 10.1523/JNEUROSCI.3977-14.2015 PMC6605500

[37] Okubo TS, Mackevicius EL, Payne HL, Lynch GF, Fee MS. Growth and splitting of neural sequences in songbird vocal development. Nature. 2015;528(7582):352–357. doi: 10.1038/nature15741 26618871 10.1038/nature15741 PMC4957523

[38] Clopath C, Büsing L, Vasilaki E, Gerstner W. Connectivity reflects coding: a model of voltage-based STDP with homeostasis. Nat Neurosci. 2010;13(3):344–352. doi: 10.1038/nn.2479 20098420 10.1038/nn.2479

[39] Bourjaily MA, Miller P. Excitatory, Inhibitory, and Structural Plasticity Produce Correlated Connectivity in Random Networks Trained to Solve Paired-Stimulus Tasks. Front Comput Neurosci. 2011;5:37 doi: 10.3389/fncom.2011.00037 21991253 10.3389/fncom.2011.00037 PMC3170885

[40] Brea J, Senn W, Pfister JP. Matching Recall and Storage in Sequence Learning with Spiking Neural Networks. J Neurosci. 2013;33(23):9565–9575. doi: 10.1523/JNEUROSCI.4098-12.2013 23739954 10.1523/JNEUROSCI.4098-12.2013 PMC6619697

[41] Rajan K, Harvey CD, Tank DW. Recurrent Network Models of Sequence Generation and Memory. Neuron. 2016;90(1):128–142. doi: 10.1016/j.neuron.2016.02.009 26971945 10.1016/j.neuron.2016.02.009 PMC4824643

[42] Tully PJ, Linden H, Hennig MH, Lansner A. Spike-Based Bayesian-Hebbian Learning of Temporal Sequences. PLoS Comput Biol. 2016;12(5):e1004954 doi: 10.1371/journal.pcbi.1004954 27213810 10.1371/journal.pcbi.1004954 PMC4877102

[43] Carr MF, Jadhav SP, Frank LM. Hippocampal replay in the awake state: a potential substrate for memory consolidation and retrieval. Nat Neurosci. 2011;14(2):147–153. doi: 10.1038/nn.2732 21270783 10.1038/nn.2732 PMC3215304

[44] Kersten D, Mamassian P, Yuille A. Object perception as Bayesian inference. Annu Rev Psychol. 2004;55(1):271–304. doi: 10.1146/annurev.psych.55.090902.142005 14744217 10.1146/annurev.psych.55.090902.142005

[45] Friston K. A theory of cortical responses. Philos Trans R Soc B Biol Sci. 2005;360:815–836. doi: 10.1098/rstb.2005.1622 10.1098/rstb.2005.1622 PMC1569488 15937014

[46] Seitz AR. Perceptual learning. Curr Biol. 2017;27(13):R631–R636. doi: 10.1016/j.cub.2017.05.053 28697356 10.1016/j.cub.2017.05.053

[47] Goodman DFM, Brette R. The brian simulator. Front Neurosci. 2009;3:192–197. doi: 10.3389/neuro.01.026.2009 20011141 10.3389/neuro.01.026.2009 PMC2751620

[48] Goodman H, Wang T, Tsodyks M. Differential signaling via the same axon of neocortical pyramidal neurons. Proc Natl Acad Sci. 1998;95(9):5323–5328. doi: 10.1073/pnas.95.9.5323 9560274 10.1073/pnas.95.9.5323 PMC20259

[49] Zucker RS, Regehr WG. Short-term synaptic plasticity. Annu Rev Physiol. 2002;64:355–405. doi: 10.1146/annurev.physiol.64.092501.114547 11826273 10.1146/annurev.physiol.64.092501.114547

[50] Froemke R, Poo MM, Dan Y. Spike-timing-dependent synaptic plasticity depends on dendritic location. Nature. 2005;434(7030):221–225. doi: 10.1038/nature03366 15759002 10.1038/nature03366

[51] Sjöström PJ, Turrigiano GG, Nelson SB. Rate, Timing, and Cooperativity Jointly Determine Cortical Synaptic Plasticity. Neuron. 2001;32(6):1149–1164. doi: 10.1016/S0896-6273(01)00542-6 11754844 10.1016/s0896-6273(01)00542-6

[52] Ibata K, Sun Q, Turrigiano GG. Rapid synaptic scaling induced by changes in postsynaptic firing. Neuron. 2008;57(6):819–826. doi: 10.1016/j.neuron.2008.02.031 18367083 10.1016/j.neuron.2008.02.031

[53] Sweeney Y, Hellgren Kotaleski J, Hennig MH. A Diffusive Homeostatic Signal Maintains Neural Heterogeneity and Responsiveness in Cortical Networks. PLoS Comput Biol. 2015;11(7):e1004389 doi: 10.1371/journal.pcbi.1004389 26158556 10.1371/journal.pcbi.1004389 PMC4497656

[54] Benda J, Herz AVM. A universal model for spike-frequency adaptation. Neural Comput. 2003;15(11):2523–2564. doi: 10.1162/089976603322385063 14577853 10.1162/089976603322385063

[55] Desai NS, Rutherford LC, Turrigiano GG. Plasticity in the intrinsic excitability of cortical pyramidal neurons. Nat Neurosci. 1999;2(6):515–520. doi: 10.1038/9165 10448215 10.1038/9165

[56] Zhang W, Linden DJ. The other side of the engram: experience-driven changes in neuronal intrinsic excitability. Nat Rev Neurosci. 2003;4(11):885–900. doi: 10.1038/nrn1248 14595400 10.1038/nrn1248

