# Supplementary material for: Bridging structure and function: A model of sequence learning and prediction in primary visual cortex
Source: PLoS Comput Biol. 2018 Jun 5;14(6):e1006187. doi: 10.1371/journal.pcbi.1006187 (PMC6003695; doi:10.1371/journal.pcbi.1006187)
Supplement: S1 Appendix — (PDF) [file pcbi.1006187.s001.pdf]

S1 Appendix: Conversion of weights to PSP amplitudes

For the neuron and synapse model we used in our study, it is to our knowledge impossible to get an analytical expression of the PSP amplitude. In addition, it is also infeasible to measure the PSP amplitude directly from the network simulation as each neuron is subject to background noise and the bombardment of many presynaptic spikes, which makes is practically impossible to extract the effect of a single PSP from the membrane potential trace. To estimate the PSP amplitudes occurring in our network, we thus took the following, different approach.

We simulated only one neuron with one incoming connection. The neuron and synapse were described by the same model as in our network model but without membrane noise and the neuron's initial membrane potential was set to the resting potential. We then simulated the neuron's response to one presynaptic spike and determined the amplitude of the PSP. We did this for different connection weights and then interpolated linearly between the resulting PSP amplitudes to get a function transforming connection weights to PSP amplitudes. In the case of the recurrent excitatory connections, the input to this function was additionally multiplied by two factors u^* and x^* before setting it as the connection weight. This was done to account for the STP, which is influencing the PSP amplitudes based on the firing history of the presynaptic neuron. u^* and x^* are the equilibrium values of the variables $u(t)$ and $x(t)$, which describe the STP. We determined u^* and x^* by setting Eqs 5 and 6 equal to zero and replacing the sum over spikes with a constant rate of 3 Hz corresponding to the average firing rate of an excitatory neuron in our model. This approach is obviously not exact but should give reasonably well estimates of the PSP amplitudes occurring in the full network.

Using connectivity data from a network that was simulated for 500 s, we transformed the weight distributions of the different connection types to the corresponding PSP amplitude distribution using weight-to-PSP transformation functions obtained via the above explained process. S1 Appendix Fig 1 shows the results for the PSP amplitude estimates in excitatory neurons. The EPSP amplitudes were, as the recurrent excitatory weights, approximately lognormal-like distributed and had a mean of 0.72 mV, similar to what was found experimentally [11,12,28]. The weights starting from inhibitory neurons and the IPSP amplitude distribution (mean of 0.96 mV) were, however, quite sharply distributed. This was a result of the way we initialized the connections involving inhibitory neurons (see Methods) and the fact that they are not subject to plasticity. The inclusion of plasticity for these kinds of connections in the LIF-SORN is currently being worked on. Finally, the distribution of the IPSP to EPSP amplitude ratios was centered around 1.

S1 Appendix Fig 2 shows the results for the PSP amplitude estimates in inhibitory neurons. The mean EPSP amplitude was 0.74 mV, the mean IPSP amplitude was 0.94 mV and the IPSP to EPSP amplitude ratios were distributed around 1.25.

To conclude, the mean PSP amplitudes in our network simulation lie within the range of experimentally observed values [11,12,28] and the EPSP amplitude distribution was shaped similarly to the experimentally observed one [12]. The distributions of IPSP amplitudes were, however, quite sharp due to our simplistic model of connections involving inhibitory neurons.

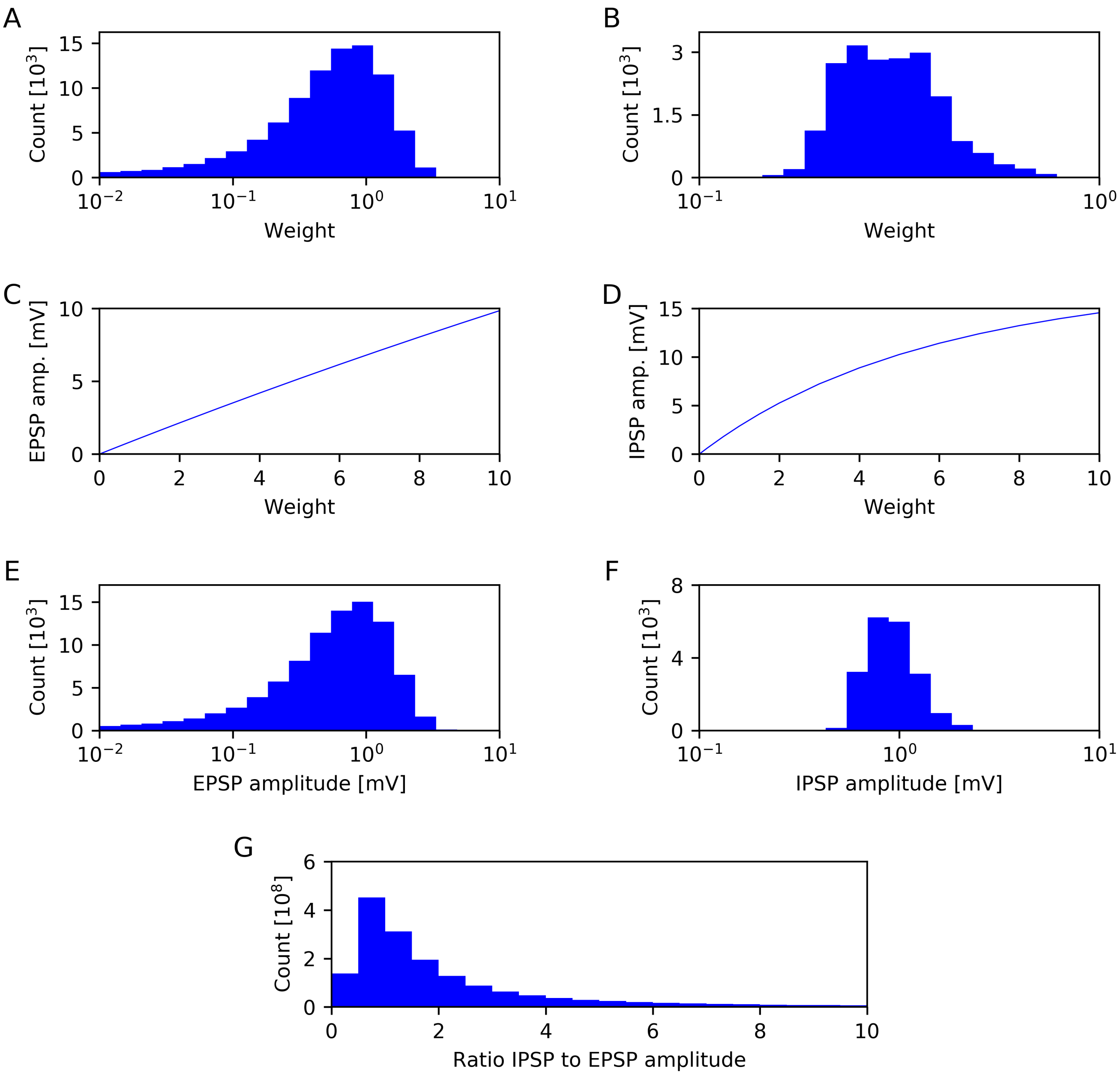

S1 Appendix Fig 1. Weights and PSPs for excitatory neurons. (A,C,E) Weight distribution (A), weight-to-PSP transformation function (C) and PSP distribution (E) for recurrent excitatory connections. (B,D,F) Same as (A,C,E) but for connections from inhibitory to excitatory neurons. (G) Distribution of ratios of IPSPs to EPSPs.

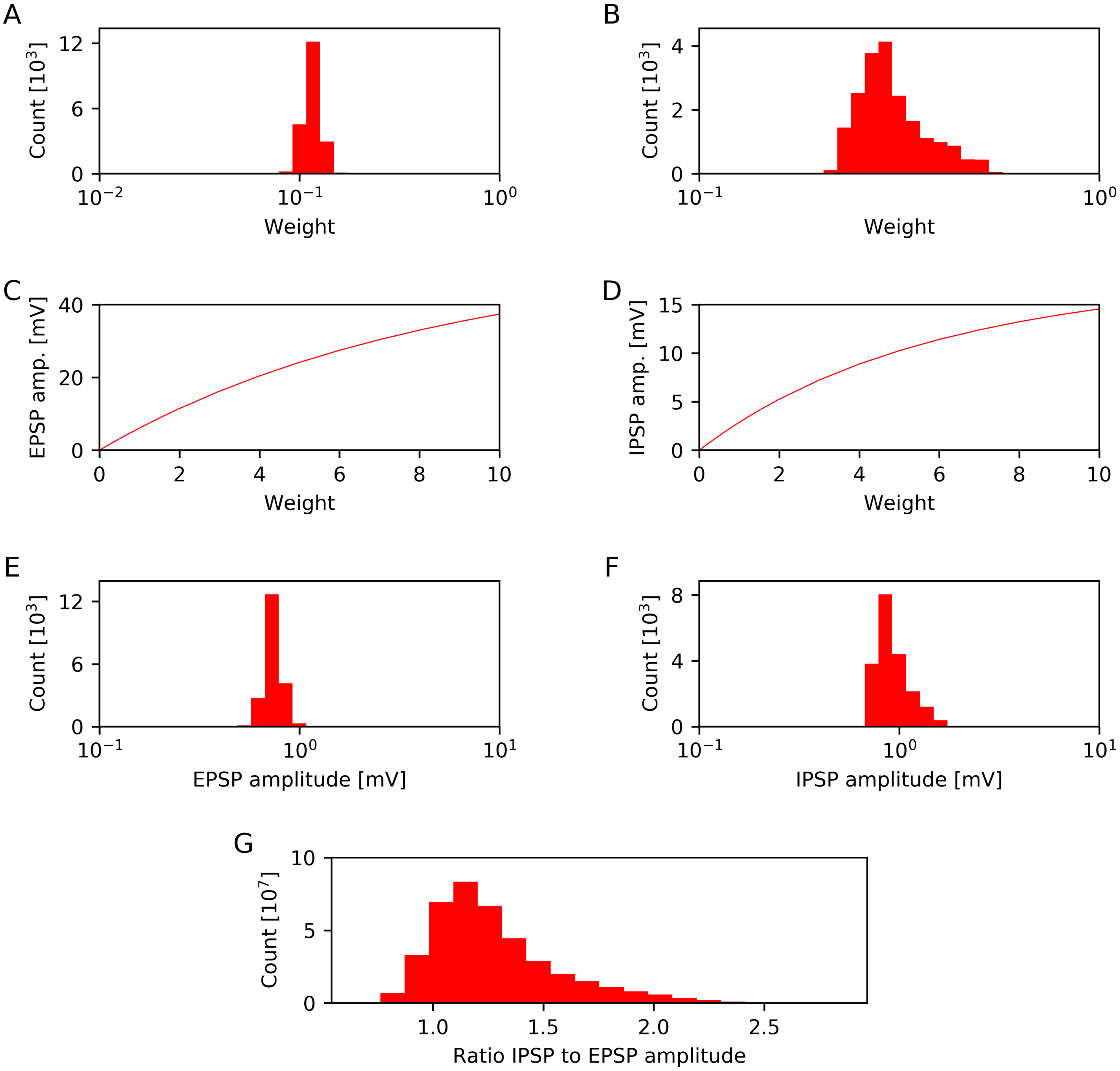

S1 Appendix Fig 2. Weights and PSPs for inhibitory neurons. (A,C,E) Weight distribution (A), weight-to-PSP transformation function (C) and PSP distribution (E) for connections from excitatory to inhibitory neurons. (B,D,F) Same as (A,C,E) but for recurrent inhibitory connections. (G) Distribution of ratios of IPSPs to EPSPs.
