# Supplementary figures and images for: Bridging structure and function: A model of sequence learning and prediction in primary visual cortex
Source: PLoS Comput Biol. 2018 Jun 5;14(6):e1006187. doi: 10.1371/journal.pcbi.1006187 (PMC6003695; doi:10.1371/journal.pcbi.1006187)

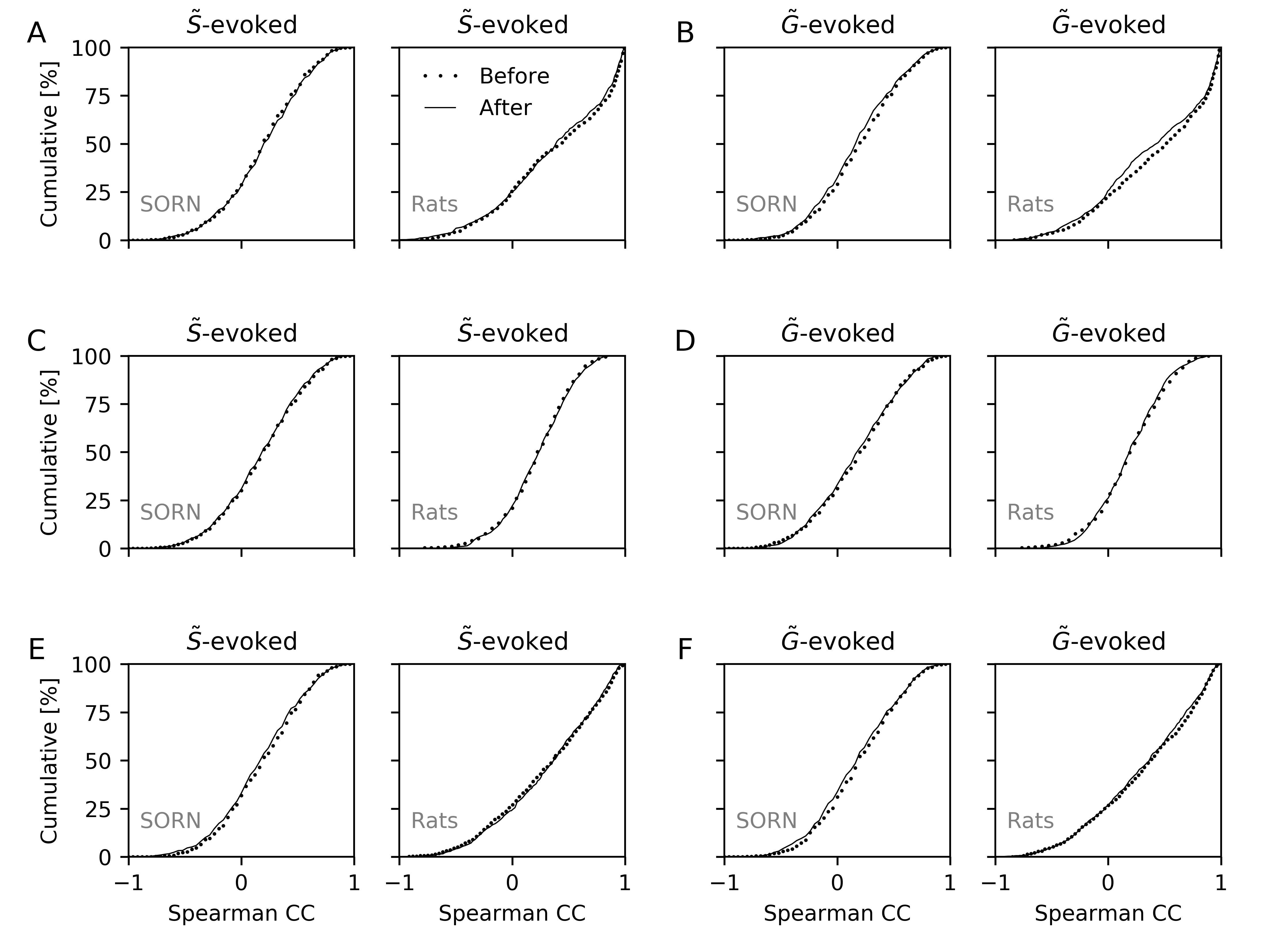

Supplement: S1 Fig — (A,B) Results for training with a moving spot whose trajectory is shifted in a direction orthogonal to the S˜→G˜ axis. In the LIF-SORN, the clusters were aligned between xS˜=(375μm,350μm)T and xG˜=(2125μm,350μm)T, while the spot moved from (375 μm, 650 μm)T to (2125 μm, 650 μm)T during training. (C,D) Results for training by flashing a bar that spans from S˜ to G˜. (E,F) Results for training by flashing a spot at S˜. Plots showing results of the LIF-SORN are based on data from 10 network instances. Plots showing results of rats were obtained from awake (bar-stimulus) and anesthetized (parallel shifted sequence, flash-stimulus) rats and were extracted from [1] using WebPlotDigitizer [30]. (TIF) [file pcbi.1006187.s002.tif]
